# Targeting the tumor microenvironment: a new strategy for natural products in breast cancer therapy

**DOI:** 10.1007/s13659-026-00641-z

**Published:** 2026-09-30

**Authors:** Yan-Cai Liu, Yan-Ran Yang, Jiang-Long Wang, Hao-Yang Yu, Min Shao, He-Feng Zhou

**Affiliations:** School of Bioengineering, Zhuhai Campus of Zunyi Medical University, Zhuhai, 519041 Guangdong People’s Republic of China

**Keywords:** Breast cancer, Tumor microenvironment, Natural products, Immunomodulation, Angiogenesis inhibition, Combination therapy

## Abstract

The development and drug resistance of breast cancer are closely associated with the heterogeneity of the tumor microenvironment (TME). Immunosuppressive cells, activated matrix components, abnormal vascular networks, and metabolic reprogramming within the TME collectively create a niche that promotes tumor survival. Despite the clinical adoption of emerging therapies such as immune checkpoint inhibitors, overall response rates remain constrained by the suppressive characteristics of the TME. Natural products, with their multi-component, multi-targeted, and low-toxicity advantages, demonstrate significant potential in reshaping the TME. This systematic review summarizes recent advances in natural products regulating the breast cancer TME. We highlight the molecular mechanisms by which natural products reverse immunosuppression, normalize tumor vasculature, inhibit stromal fibrosis, and correct metabolic dysregulation. Additionally, synergistic strategies combining natural products with chemotherapy and immunotherapy are explored, particularly how nanotechnology-based delivery systems overcome pharmacokinetic barriers. Finally, we critically analyze translational challenges in this field and outline future research directions.

**Graphical abstract:**

**Figure:**
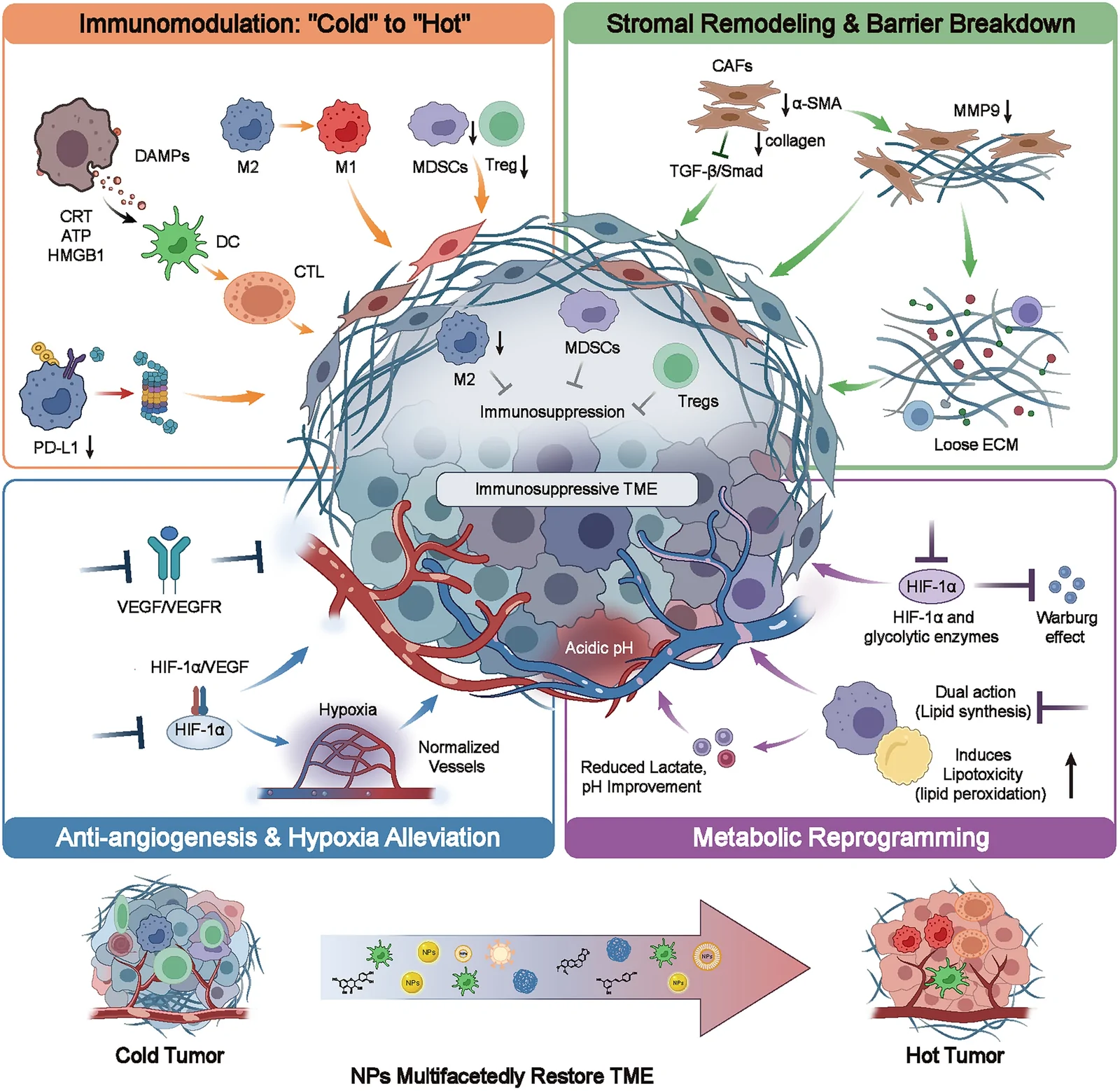

## Introduction

Breast cancer is a leading cause of cancer death in women globally [1]. Treatments like surgery, chemotherapy, radiotherapy, and targeted therapy have significantly helped patients live longer. However, problems like metastasis, recurrence, and multidrug resistance (MDR) are still hard to solve in the clinic [2–5]. Modern cancer research suggests that a tumor is not just an isolated group of cells. Instead, it is an ecosystem that develops together with the complex TME. The TME drives the progression of breast cancer by suppressing the immune system, inducing epithelial-mesenchymal transition (EMT), and maintaining stem cell properties [6–10]. Therefore, the focus of breast cancer treatment is shifting from just attacking tumor cells to a new strategy of remodeling the tumor soil [11, 12].

Natural products (NPs) are a valuable source for drug discovery. They possess diverse chemical structures and can target multiple signaling pathways simultaneously. This property is known as polypharmacology, and it gives NPs a distinct advantage in regulating the TME [13]. Unlike drugs that act on a single target, NPs such as polyphenols, alkaloids, and saponins work through synergistic mechanisms. They can simultaneously regulate the immune environment, inhibit angiogenesis, and improve metabolic status [13–21]. With the development of nanomedicine and multi-omics technologies, the bioavailability and targeting ability of NPs have improved significantly. These advances provide new momentum for their clinical translation [22, 23]. This article aims to review the latest research on how NPs remodel the breast cancer TME. We focus on their regulatory effects in four key areas: immunity, stroma, blood vessels, and metabolism. Finally, we discuss the prospects and challenges of using NPs as part of combined treatment strategies.

## The landscape of breast cancer TME

The TME of breast cancer is a highly heterogeneous and dynamic ecosystem. Its composition and functional state directly determine the malignant features and clinical outcomes of the tumor [24]. The TME is not merely a passive support structure for tumor cells. Instead, it actively builds a "shelter" that favors immune escape and drug resistance through complex cell communication and molecular interactions [25, 26]. This microenvironment is mainly maintained by immunosuppressive cells, activated stromal components, and abnormal physical and chemical conditions such as hypoxia and metabolic reprogramming.

### The construction of the immunosuppressive network and therapeutic escape

The profile of immune cells in the TME is key to the effectiveness of the anti-tumor immune response [27]. Effector T cells and natural killer (NK) cells try to perform immune surveillance. However, the TME is often enriched with large numbers of suppressive cell groups. These include tumor-associated macrophages (TAMs), myeloid-derived suppressor cells (MDSCs), and regulatory T cells (Tregs). Together, these cells form a barrier that allows the tumor to escape the immune system [28]. TAMs are the most abundant immune cells in the TME. They are often reprogrammed into the pro-tumor M2 phenotype. They restrict the function of CD8 + T cells by depositing collagen and remodeling the metabolic environment [29]. Notably, recent studies have revealed more complex interactions between cells. Cancer-associated fibroblasts (CAFs) can induce the formation of lipid-associated macrophages. These cells work together to mediate immune suppression [30]. In addition, tumor-derived arachidonic acid can reprogram neutrophils to acquire inhibitory functions and lead to therapeutic resistance [31]. The TME can even hinder immune attacks through physical mechanisms. For example, CAFs have been found to act as "decoys" that directly exhaust the cytotoxicity of NK cells[32]. In specific contexts like CDK4/6 inhibitor treatment, the interaction between IL-17A-secreting γδ T cells and CX3CR1 + macrophages has been confirmed as a key mechanism leading to drug resistance [33].

### Stromal remodeling and physical barriers

Stromal cells, especially CAFs, are the main force driving the structural remodeling of the TME. Single-cell sequencing technology has revealed that CAFs have high heterogeneity in their spatial distribution and functions [34, 35]. CAFs support tumor growth by secreting cytokines. They also cause tissue stiffening by synthesizing excessive amounts of extracellular matrix(ECM) [36]. This stiff physical environment enhances the ability of the tumor to invade nerves through integrin signaling pathways [37, 38]. Furthermore, the arrangement of collagen fibers is closely associated with poor prognosis [39]. Studies on functional subpopulations show that TSPAN8 + myofibroblastic CAFs directly promote chemotherapy resistance [40]. In addition, CAFs can regulate endothelial cell function through paracrine signaling [41]. They may remodel the perivascular venular niche to specifically controllymphocyte extravasation. This process limits the infiltration of immune cells into the tumor core [42]. Moreover, a complex network of cytokines, such as the chemokine C‑C motif chemokine ligand 2 (CCL2), further promotes tumor metastasis and malignant progression [43].

### Hypoxia, metabolic reprogramming, and angiogenesis

The rapid proliferation of tumor cells leads to a relative shortage of oxygen and nutrients. This creates the unique physical and chemical characteristics of the TME. A continuous hypoxic environment stabilizes hypoxia-inducible factor-1α (HIF-1α). This drives the high expression of vascular endothelial growth factor (VEGF). Consequently, it leads to the formation of tumor blood vessels that are structurally abnormal and highly leaky. This process not only worsens hypoxia but also promotes the formation of vasculogenic mimicry (VM). VM does not rely on endothelial cells and provides channels for tumor metastasis [44]. At the same time, hypoxia forces tumor cells to undergo metabolic reprogramming, known as the "Warburg effect." This produces a large amount of lactate and causes acidification of the microenvironment. This acidic environment has been proven to directly inhibit T cell activity and promote the polarization of M2 macrophages [45]. In addition to glucose metabolism, lipid metabolic reprogramming is also crucial [46]. Metabolic crosstalk occurs between tumor cells, adipocytes, and other stromal cells. Specifically, the transfer and utilization of lipids provide key energy support for tumor cells [47]. Furthermore, tumor-specific metabolites such as N-acetylaspartylglutamate have been demonstrated to remodel the TME to promote metastasis [48]. There is also close crosstalk between hypoxia and the ECM [49], Moreover, targeting key metabolic enzymes like PGAM1 has been proven effective in remodeling the TME and enhancing the efficacy of immunotherapy [50].

### Subtype-specific TME heterogeneity and differential natural product interventions

Although immunosuppressive networks, stromal barriers, hypoxia, and metabolic dysregulation are common features of breast cancer, the TME is not a uniform entity. Increasing evidence from multi-omics analyses indicates that the composition, spatial organization, and functional state of the TME vary substantially across molecular subtypes of breast cancer [51]. This subtype-dependent heterogeneity shapes distinct patterns of immune evasion, therapy adaptation, and metastatic progression, suggesting that NP-based interventions should not be viewed as identical strategies. Instead, their polypharmacological properties may be particularly valuable when aligned with subtype-specific vulnerabilities, including endocrine-stromal interactions, innate immune dysfunction, angiogenic dependence, and metabolic addiction [17, 52].

#### Luminal (HR +) breast cancer

Luminal breast cancers are generally considered relatively “immune-cold,” with limited lymphocytic infiltration and weaker baseline immunogenicity than more aggressive subtypes. Their TME is strongly shaped by estrogen receptor (ER)-dependent signaling, which coordinates a complex endocrine-stromal crosstalk that supports tumor persistence and therapeutic adaptation [53]. In addition, oncogenic alterations frequently observed in this subtype, such as PIK3CA mutations, may further reinforce immunosuppression by promoting myeloid-derived suppressor cell (MDSC) recruitment through arachidonic acid-related pathways [54].

A major therapeutic challenge in luminal disease is the emergence of endocrine resistance, including resistance to tamoxifen and related agents. This process is increasingly recognized as a microenvironment-associated event, closely linked to hypoxia, altered energy metabolism, and stromal support [5]. Accordingly, NPs that disrupt endocrine-stromal communication or reverse resistance-associated metabolic adaptation may be particularly relevant in this setting. For example, baicalein has been reported to resensitize tamoxifen-resistant breast cancer cells by suppressing the HIF-1α axis, thereby reducing aerobic glycolysis and improving mitochondrial dysfunction [55]. These findings suggest that, in luminal breast cancer, NPs may act not primarily as broad immune activators, but as modulators of endocrine resistance and chronic stromal-metabolic support.

#### HER2-enriched breast cancer

The HER2-enriched subtype is commonly associated with a highly angiogenic microenvironment driven by oncogene-induced upregulation of pro-angiogenic programs. In this context, NPs with anti-angiogenic activity may help attenuate vascular abnormalities and reduce microenvironmental support for tumor progression. Emodin, for instance, has been shown to inhibit angiogenesis in breast cancer by suppressing VEGFA transcription [56].

Beyond angiogenesis, the therapeutic efficacy of anti-HER2 agents such as trastuzumab depends in part on NK cell-mediated antibody-dependent cellular cytotoxicity (ADCC) [57, 58]. However, this innate immune mechanism can be compromised by suppressive stromal components within the TME. Notably, PDPN-positive CAFs have been reported to impair NK cell-mediated ADCC and contribute to trastuzumab resistance [59, 60]. Therefore, restoration of innate cytotoxic immunity represents a particularly relevant strategy in HER2-positive disease. Consistent with this concept, Xianling Lianxia formula has been reported to enhance trastuzumab efficacy in HER2-positive breast cancer by improving NK-cell function [61]. Taken together, these observations suggest that NP-based strategies in HER2-enriched tumors may be especially useful when they combine anti-angiogenic effects with reinforcement of trastuzumab-dependent innate immune surveillance.

#### Triple-negative breast cancer

Triple-negative breast cancer (TNBC) presents a marked immunological paradox. Although this subtype often exhibits higher baseline immunogenicity and increased tumor-infiltrating lymphocytes (TILs), its clinical outcome remains poor because of the rapid evolution of immune escape mechanisms [62, 63]. Current evidence indicates that TNBC-associated immunosuppression is sustained by a complex cellular network involving M2-polarized TAMs,MDSCs, and Tregs [64, 65]. In parallel, CAF-mediated ECM remodeling generates a dense desmoplastic barrier that not only physically restricts immune-cell infiltration but also promotes an immune-excluded phenotype through biochemical signaling [66, 67]. Accordingly, effective TME-directed strategies for TNBC should emphasize the selective dismantling of suppressive cellular circuits and stromal barriers rather than relying solely on nonspecific immune activation [68].

**Fig. 1:**
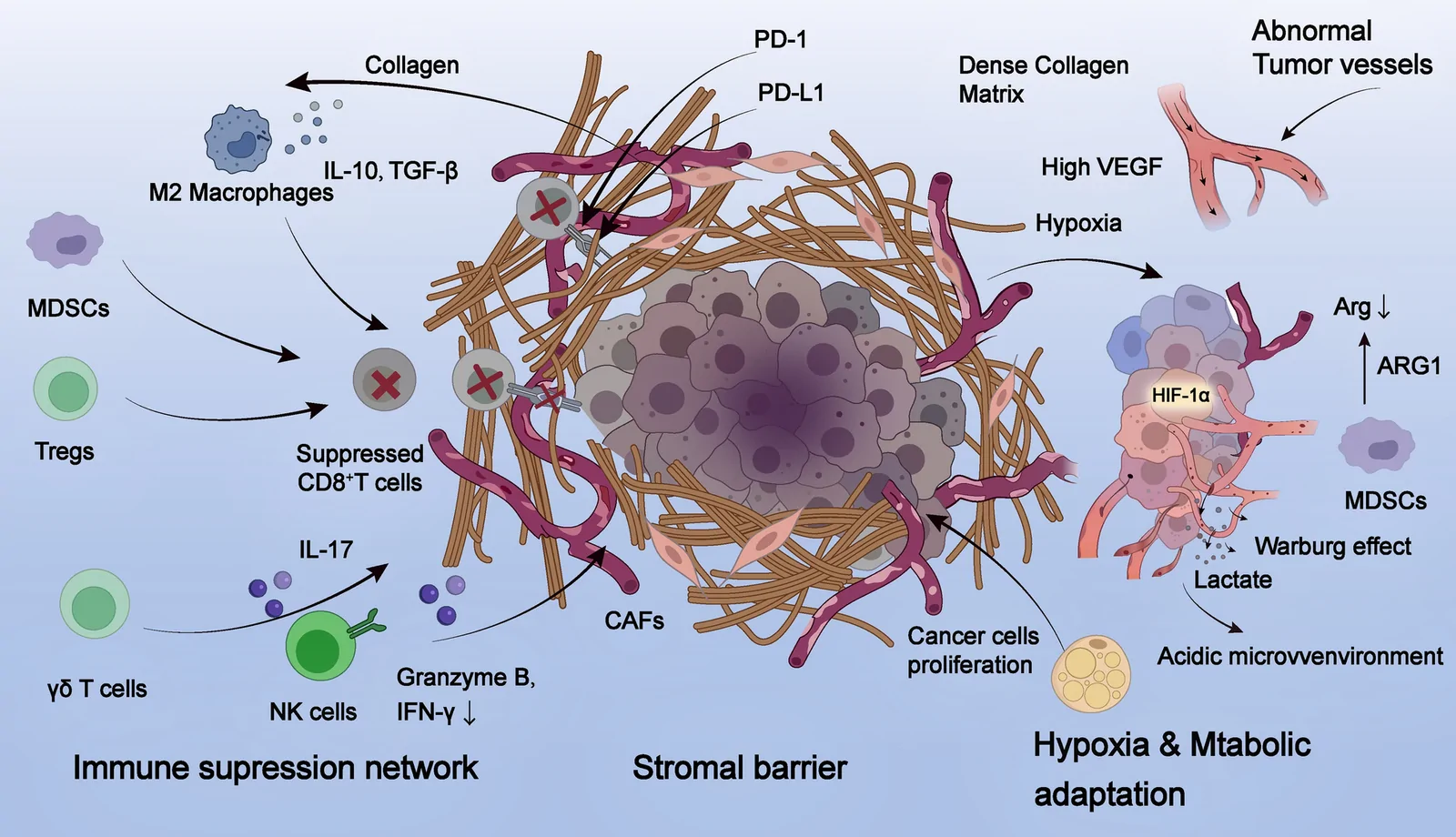
Overview of the breast cancer tumor microenvironment

Importantly, this immune-tolerant state is mechanistically intertwined with the metabolic rewiring of TNBC cells. To sustain rapid proliferation and maintain redox balance, TNBC cells display increased dependence on specific amino acids, particularly glutamine and arginine [69, 70]. By upregulating amino acid transporters such as SNAT2/SLC38A2, tumor cells compete aggressively for nutrients within the local microenvironment and adapt dynamically to metabolic stress [71, 72]. Such nutrient competition can result in severe metabolic deprivation in the TME, which in turn drives T-cell dysfunction, metabolic exhaustion, and impaired anti-tumor immunity [73, 74]. Therefore, compared with generalized immune stimulation, NP-based interventions that simultaneously target immunosuppressive circuits and metabolic addiction may offer greater specificity and translational relevance for restoring TME homeostasis in TNBC（Fig.1；Table1）.

## Remodeling the immune microenvironment: from "cold" to "hot" tumors

Transforming "cold tumors" that lack immune cell infiltration into inflammatory "hot tumors" is a crucial strategy to overcome the current challenges in breast cancer immunotherapy [75–79]. NPs possess polypharmacology characteristics, including multi-target effects and low toxicity. Therefore, they demonstrate unique advantages in breaking immune tolerance within the TME. Unlike synthetic drugs that focus on a single target, NPs can systematically rebuild the anti-tumor immune cycle. They achieve this through multi-dimensional mechanisms such as inducing immunogenic cell death (ICD), reprogramming immune cell metabolism, and degrading immune checkpoints.

### Inducing ICD and enhancing antigen presentation

Traditional chemotherapy often induces poorly immunogenic forms of tumor cell death, allowing tumor antigens to remain immunologically silent. In contrast, a number of natural NPs can trigger ICD, a form of regulated cell death that converts dying breast cancer cells into a source of endogenous danger signals[80, 81] . Hallmarks of ICD include the exposure of calreticulin (CRT) on the cell surface, the release of ATP, and the secretion of HMGB1, all of which promote the recruitment and activation of dendritic cells (DCs)p [82] . In breast cancer models, compounds such as oleandrin, Cordyceps militaris extract, and marine-derived small molecules have been shown to induce ICD-associated molecular events, thereby increasing the immunogenic visibility of tumor cells [83–85]. Likewise, pterostilbene and Ganoderma lucidum extract can induce inflammatory forms of tumor cell death and remodel the tumor immune microenvironment, further supporting the conversion of immune-desert tumors into inflamed lesions [80, 86]

**Table 1:** Subtype-specific TME features in breast cancer

Breast cancer subtype	Dominant TME features	Major therapeutic barriers	Natural product activities likely to be most relevant
TNBC	Immune-infiltrated but strongly immunosuppressed; abundant TAMs/MDSCs/Tregs; dense CAF/ECM remodeling; hypoxia, glycolysis, lactate accumulation	T-cell exhaustion, NK dysfunction, immune exclusion, stromal barrier, metabolic immune suppression, rapid metastasis	Macrophage repolarization, MDSC reduction, PD-L1/immune escape inhibition, anti-hypoxia, anti-glycolysis, ECM/CAF normalization
Luminal A/B	Immune-cold or immune-restricted; chronic stromal support; fibrotic ECM; endocrine resistance-associated immune and metabolic adaptation	Low immunogenicity, poor immune infiltration, fibrotic barrier, endocrine resistance, therapy-adaptive metabolism	Anti-fibrotic and anti-CAF effects, metabolic adaptation control, modulation of chronic myeloid suppression, endocrine resistance-associated TME reprogramming
HER2-positive	Intermediate-to-high immune activity; partial dependence on NK-cell-mediated ADCC; stromal-vascular remodeling; treatment-induced adaptive resistance	Impaired NK-cell function, CAF-mediated resistance, abnormal vasculature, reduced efficacy of anti-HER2 therapy	NK-cell enhancement, stromal-vascular normalization, relief of CAF-mediated immune suppression, anti-angiogenic modulation

Beyond sensing DAMPs, DCs are the central professional antigen-presenting cells that connect innate immune activation to adaptive anti-tumor immunity. However, in breast cancer, DCs frequently remain functionally impaired or insufficiently matured within the immunosuppressive TME, which limits antigen uptake, co-stimulatory signaling, and subsequent T-cell priming [87]. NPs can help overcome this bottleneck by promoting DC maturation and improving antigen presentation. For example, polysaccharides derived from Plantago asiatica and Portulaca oleracea have been reported to enhance the efficacy of DC-based immunotherapy in breast cancer, while Ganoderma lucidum polysaccharide-based systems stimulate DC activation and memory T-cell responses [88–90] Mechanistically, these effects are associated with increased expression of co-stimulatory molecules and improved antigen-presenting capacity, thereby facilitating the cross-priming of tumor-specific CD8 + T cells. In parallel, activation of the cGAS-STING/type I interferon axis by certain NPs, including Schisandrin C and fucoidan-based formulations, may further reinforce DC recruitment, maturation, and antigen cross-presentation, thus amplifying the crosstalk between innate and adaptive immunity and creating a cytokine milieu favorable for effector T-cell infiltration [91, 92]. Collectively, these findings indicate that NPs not only induce ICD at the tumor-cell level but also strengthen the downstream DC-centered antigen-presentation cascade, a critical step in driving the transition from “cold” to “hot” breast tumors.

### Reprogramming tumor-associated macrophages

TAMs typically exhibit a pro-tumor M2 phenotype within the TME, establishing an immunosuppressive barrier by secreting IL-10 and transforming growth factor β(TGF-β). Reversing TAMs from the M2 phenotype to the tumor-killing M1 phenotype is a core target for natural product interventions. Compounds such as Taraxacum mongolicum can block key signaling axes like STAT3 or PI3K/Akt/mTOR. This compels TAMs to polarize toward the M1 phenotype, restoring their phagocytic function and ability to secrete pro-inflammatory cytokines (such as TNF-α and IL-12) [93–96]. Similarly, Glycyrrhetinic acid and extracts of Smilax china L. activate the JNK1/2 and ERK1/2 signaling pathways, respectively. This promotes the reversion of M2 macrophages to the M1 type, thereby blocking metastasis and enhancing the anti-tumor immune response [97, 98]. Additionally, Lycium barbarum polysaccharides (LBP) of specific molecular weights have demonstrated the ability to induce tumor regression by repolarizing TAMs [99]. This reprogramming effect not only reduces the production of suppressive mediators but also remodels the ECM by regulating the secretion of matrix metalloproteinases (MMPs). This process clears physical obstacles for the infiltration of effector T cells [100, 101].

### Degrading checkpoints and relieving suppression

Blocking the programmed cell death protein-1(PD-1)/programmed death-ligand 1(PD-L1) pathway is the dominant strategy in current clinical immunotherapy. However, many natural products provide an alternative approach beyond antibodies. They can directly lower the expression levels of checkpoint proteins. Antibodies primarily block the binding between receptors and ligands. In contrast, natural small molecules tend to exert their effects at the level of post-translational modifications. Recent studies have found that Berberine derivatives and Resveratrol can interfere with the glycosylation of PD-L1. This leads to protein misfolding and retention in the endoplasmic reticulum. Alternatively, these compounds can directly promote ubiquitination. This process causes PD-L1 to be degraded through the proteasome pathway [19, 102]. This mechanism reduces PD-L1 abundance on tumor cell surfaces at its source, effectively lifting T cell inhibition. Furthermore, targeting MDSCs—another critical inhibitory cell population in the TME—substances like Grifola frondosa polysaccharide (GFI) have been shown to induce their apoptosis or promote their differentiation into mature myeloid cells, thereby further clearing immune brake mechanisms within the TME [103, 104].

### Restoring innate and innate-like cytotoxic immunity

NPs may further enhance anti-tumor immunity by restoring innate and innate-like cytotoxic lymphocytes, particularly NK cells and γδ T cells. In breast cancer, NK-cell function is frequently suppressed by the TME, resulting in reduced cytotoxicity and impaired IFN-γ production [32, 60, 105–107]. Several NPs have been reported to counteract this dysfunction; for example, tanshinol reverses TGF-β1-mediated NK-cell suppression, while Maitake β-glucan enhances trastuzumab efficacy by strengthening NK cell-mediated ADCC [108, 109]. In addition, black rice bran polysaccharides and Pereskia bleo extracts have shown the ability to augment NK-cell activity in TNBC-related models [110, 111]. γδ T cells may also contribute to this process because of their rapid, largely MHC-independent cytotoxic potential, although their functions in breast cancer are heterogeneous and context dependent, with some subsets exerting anti-tumor effects while IL-17-producing γδ T cells may instead promote metastasis and immunosuppression [33, 112–118]. Available evidence suggests that certain natural molecules may help preserve γδ T-cell fitness and partially restore cytotoxic activity under suppressive conditions [119, 120]. Although this area remains less explored, these findings indicate that modulation of NK cells and γδ T cells may represent an additional mechanism by which NPs help convert breast tumors from immunologically “cold” to more responsive states.

## Targeting stroma and inhibiting angiogenesis: overcoming physical barriers

Beyond the immunosuppressive microenvironment, breast cancer (especially triple-negative breast cancer) is typically characterized by significant desmoplasia and disordered vascular structure. This abnormal physical barrier not only directly limits the infiltration of chemotherapeutic agents and immune effector cells into the tumor core, but also leads to high interstitial fluid pressure (IFP) and sustained hypoxia, which in turn induce therapeutic resistance and metastasis [66, 121–124]. Therefore, using natural products to achieve "TME normalization"—specifically through matrix softening and vascular repair—has become a highly promising therapeutic strategy.

### Inhibiting CAF activation and matrix softening

CAFs are the primary source of the ECM in the TME. Driven continuously by the TGF-β/Smad signaling pathway, CAFs secrete excessive amounts of collagen and fibronectin, constructing a dense physical barrier. Various natural compounds have demonstrated significant anti-fibrotic activity. Furthermore, various flavonoids can regulate the balance between MMPs and their tissue inhibitors (TIMPs), promoting the degradation of pathologically deposited matrix [125]. This "matrix softening" effect significantly reduces the stiffness and interstitial pressure of the tumor tissue, opening physical channels for chemotherapeutic agents to reach deep tumor tissues [126–128]. Recent studies have also focused on Halofuginone, suggesting it may block collagen synthesis by specifically inhibiting prolyl-tRNA synthetase, thereby demonstrating excellent matrix remodeling capabilities in breast cancer models [129–131].

### Anti-angiogenesis and alleviating hypoxia

Targeting abnormal tumor angiogenesis and alleviating the resulting hypoxia is an effective strategy to remodel the physical microenvironment of breast cancer. Natural products exert anti-angiogenic effects through multiple pathways, among which inhibiting VEGF and its downstream signaling pathways is a core mechanism. For example, Emodin can specifically target the transcriptional process of VEGFA, thereby significantly inhibiting breast cancer angiogenesis and disrupting the tumor's blood supply [56]. Isoliquiritigenin inhibits the VEGF angiogenic factor, blocking the formation of new tumor blood vessels [132]. Apigenin can inhibit tumor angiogenesis by reducing VEGF levels [133]. On the other hand, correcting the hypoxic state in the TME and blocking the pro-survival and pro-resistance adaptive responses driven by HIF-1α is crucial for reversing malignant progression. As a key transcription factor in the cellular response to hypoxia, HIF-1α broadly regulates the expression of a series of genes involved in angiogenesis and metabolic reprogramming. Multiple studies indicate that natural products can effectively intervene in this pathway. Phytochemicals in Moringa oleifera can block the adaptive survival of breast cancer cells in hypoxic environments by inhibiting the HIF-1α/VEGF/GLUT1 signaling axis [134]. Sanguinarine can simultaneously inhibit the hypoxia-induced activation of EphB4 and HIF-1α pathways and interfere with hypoxia signal transduction at the transcriptional level by disrupting the interaction between HIF-1α and phosphorylated STAT3 [135, 136]. Additionally, Plumbagin can downregulate HIF-1α protein expression under hypoxic conditions via a non-PI3K/Akt/mTOR-dependent pathway, demonstrating a unique mechanism for hypoxia reversal [137].

## Metabolic reprogramming: severing energy supply

Tumor cells exhibit significant metabolic plasticity, characterized specifically by enhanced aerobic glycolysis (the Warburg effect) and abnormal lipid metabolism. These metabolic alterations not only provide energy and biosynthetic building blocks for rapid tumor proliferation but also lead to lactate accumulation and acidification of the TME, which in turn inhibits anti-tumor immunity and promotes metastasis [138]. Unlike synthetic metabolic inhibitors that mainly target a single enzyme, natural products employ multi-dimensional target interventions. They can both cut off the energy supply of tumor cells and correct the metabolic imbalance of the TME, thereby achieving normalization of the immune microenvironment at the metabolic level.

### Targeting the warburg effect and ameliorating the acidic niche

Aerobic glycolysis is a core metabolic mode for breast cancer to adapt to hypoxic environments and maintain malignant proliferation. HIF-1α, as a key transcription factor driving the Warburg effect, is a primary target for natural products to ameliorate TME acidification. Cryptolepine and Coenzyme Q0 have been confirmed to significantly inhibit HIF-1α expression thereby blocking the expression of downstream glycolytic enzymes. This not only curbs tumor growth but also simultaneously inhibits NLRP3-mediated inflammatory responses and the EMT process [139, 140]. In addition to transcriptional regulation, natural products can directly exert allostericregulation on key rate-limiting enzymes. Cryptanshinone blocks glycolysis via the pyruvate kinase M2 (PKM2)/β-catenin axis to inhibit cell migration [141], while the flavonoid compound GL-V9 disrupts the binding of Hexokinase II (HKII) to mitochondria (regulated by GSK-3β), relieving the inhibition of apoptosis by HKII [142]. Naringin may remodel the functional phenotype of TAMs at the metabolic level by regulating the glycolytic rate [143]. This systemic intervention targeting the glycolytic pathway effectively reduces lactate secretion, reversing the TME from an acidic suppressive state to a physiological state favorable for T cell survival.

### Dual intervention of lipid metabolism: blocking synthesis and inducing toxicity

Lipid metabolic reprogramming provides biomembrane components and signaling molecules for tumor cells, acting as another major engine driving breast cancer metastasis. Natural products adopt a unique "dual intervention" strategy in this regard. On one hand, to cut off the tumor's lipid supply, Compound Kushen Injection disrupts cell membrane stability by dual targeting of the lipogenesis pathway and polyunsaturated fatty acid (PUFA) homeostasis, thereby effectively inhibiting breast cancer bone metastasis [144] (Table 2). On the other hand, exploiting the sensitivity of tumor cells to reactive oxygen species (ROS), some natural products exert anti-cancer effects by inducing "lipotoxicity." For example, bound polyphenols derived from the inner shell of foxtail millet trigger lipid peroxidation-mediated autophagic cell death by promoting excessive intracellular lipid accumulation [191]. This strategy cleverly transforms lipid metabolism from a survival advantage into a fatal defect for the tumor.

**Table 2:** Natural product-mediated regulation of the TME

No.	Type	Natural products	Chemical structure	Source	Cell lines or animal models	Mechanism of action	Result	Refs.
1	Polyphenols	Resveratrol		N/A	NK92、K562	Akt↑ mTORC2↑c-Myb↑	NK cells↑	[145]
2	Terpenoids	Isotoosendanin		*Melia toosendan *	MDA-MB-231、BT549、4T1; BALB/c	Targeting TGFβR1,TGFβR1 kinase↓	EMT↓ M1/M2↑anti-PD-L1↑ CD3⁺ T↑CD8⁺ T↑ Granzyme B⁺ T↑CD107a⁺ T↑ collagen deposition↓DC↑	[146]
3	Terpenoids	Triptonide		*Tripterygium wilfordii* Hook F	MDA-MB-231、MDA-MB-468	Twist1 ↓Notch1 ↓ p-NF-κB↓	N-cadherin↓ VE-cadherin↓ VEGFR2↓ angiogenesis ↓vasculogenic mimicry↓	[147]
4	Polyphenols	Icariin		*Epimedium L. wushanense* Maxim, *Epimedium sagittatum* maxim, *Epimedium pubescens* maxim	MDA-MB-231、4T1; BALB/c,BALB/c nude	SIRT6↑p-IκBα↓NF-κB p65↓	E cadherin↑ N cadherin↓ MMP 2↓PD L1↓ CD4⁺/ CD8⁺ T↑MDSCs↓	[148]
							CD8⁺ T↑MDSCs↓	
5	Saponins	Anemoside A3		*Pulsatilla chinensis*	4 T1-Luciferase(Luc)、L929、BMDM;C57BL/6,BALB/c	p-STAT3↓ p-JAK2-	M2↓CCL2↓VEGF↓CCL7↓ MMP-9↓	[149]
6					MCF-7、4T1-Luc、THP-1;BALB/c	Toll-Like Receptor4/NF-κB/MAPK↑	M1↑angiogenesis↓CD86+↑ TNF-α↑IL-12↑ VEGF↓	[150]
7	Quinones	emodin		*Rheum palmatum* L	MDA-MB-231、4T1、4T1-Luc; BALB/c 、NOD-SCID, BALB/c,zebrafish	targeting NCOR2, SerRS↑VEGFA↓M2↓M1↑	angiogenesis↓anti-tumor immunity↑	[56]
8	Polyphenols	Anhydroicaritin		*Epimedium brevicornu* Maxim Cullen corylifolium (L.) Medik.	MCF-7、SK-BR-3、4T1、 MDA-MB-231;BALB/c	HIF-1α↓VEGFA↓MMP2↓	metastasis↓	[151]
9	Alkaloids	Berberine		*Coptis chinensis* Franch	4T1、4T1/Luc、 MDA-MB-231、MCF-7;BALB/c	HIF-1α↓ **Location in the manuscript: **Table 2, Row 10 (E-cadherin). Correction detail: Change the arrow direction next to E-cadherin to point upward (↑). **Reason for change: **To ensure the accuracy of the data presented in Table 2. regulated endogenous metabolites	metastasis↓	[152]
10	Saponins	Saikosaponin A		*Bupleurum chinense* DC	DMBA-induced mammary carcinoma （SD）rats	IL-12/STAT4↑Th1↑Th2↓ CD8⁺ T↑CD4 + T↑	antitumor immunity↑	[153]
11	Terpenoids	Artemisinin		*Artemisia annua* L	4T1;BALB/c	TGF-β ↓ %MDSCs↓%Tregs↓ CD4⁺ IFN-γ⁺ T↑CTLs↑ IFN-γ↑TNF-α↑T-bet↑	anti-tumor immune response↑	[154]
12	Terpenoids	Artesunate; dihydroartemisinin		*Artemisia annua* L	L-929-CAFs、4T1; BALB/c,MMTV-PyMT	TGF-β1/Smad3↓	CAFs↓ metastasis↓	[155]
13	Saponins	Ginsenoside Rh2		*Panax ginseng* C. A. Meyer	MDA-MB-231、4T1、NK92MI NK cells、MCF-7;BALB/c，BALB/c nude	ERp5↓ NKG2D-MICA↑	NK cells↑	[156]
14	Polysaccharides	Solanum nigrum Linne polysaccharide-I(SNLP-1)	N/A	*Solanum nigrum* L	RAW264.7, C57BL/6	TLR4-MyD88↑	the release of NO and TNF-α and IL-6 secretion in macrophages↑	[157]
							Th1 responses mediated by IL-2, IFN-γ, and TNF↑	
							the spleen index, thymus index↑	
15	Meroterpenoid	MZ58		*Salvia miltiorrhiza* Bunge	MDA-MB-231、Jurkat T、MC38;mouse primary T cells、 C57BL/6J	IFN-γ↑ Granzyme B↑TNF-α↑PD-1/PD-L1↓	CD8 ^+^ T↑CD4^+ ^T cell exhaustion↓	[158]
16	Xanthones	α-mangostin		*Garcinia mangostana* L	REC、T-47D、MDA-MB-231; BJMC3879、BJMC3879luc2	VEGF↓p-VEGFR-2↓tumor microvessel density↓	angiogenesis↓	[159]
						PD-L1↓		
17	Polyphenols	Tanshinol		*Salvia miltiorrhiza* Bunge	NK92MI 、ZR-75–1; Balb/c nude (ZR-75–1)	NKG2D-DAP10↑TGF-β1/SMAD↓ PI3K–ERK–PLCγ2↑	TGF-β1↓lung metastasis↓	[108]
							serum levels of IFN-γ and perforin↑	
							NK cell↑CD107a↑	
18	Terpenoids	Platycodin D		*Platycodon grandiflorus* (Jacq.) A. DC	4 T1、HL-60、dHL-60、primary neutrophils;Balb/c	PD-L1↓ PI3K/Akt↓	pulmonary metastasis↓	[160]
19	Polysaccharides	polysaccharide isolated from Coriolus versicolor	N/A	*Trametes versicolor* (L.) Lloyd	RAW264.7、4T1;BALB/c	TLR4/Akt/JNK/NF-κB↑TREM2↓	M2↓M1↑ CD8^ +^ T↑	[161]
20	Complex mixture	Polysaccharide-rich extract from polygonatum sibiricum	N/A	*Polygonatum sibiricum* Redouté	4T1;BALB/c	G-CSF↓HSPCs(LSK) 、 LT-HSCs、CLPs↑WBC、MDSCs、T cells↓ Proportion of TAMs and MDSCs (PMN-MDSC, M-MDSC) ↓	protected hematopoiesis in bone marrow	[162]
21	Polysaccharides	Plantain polysaccharide	N/A	*Plantago asiatica* L	4T1、C57BL/6,CIPp; BALB/c 、BALB/c nude	CD80、CD86、MHC II↑	DCs↑immune cells↑	[163]
22	Quinones	Dihydrotanshinone I		*Salvia miltiorrhiza* Bunge	MDA-MB-231、4T1、 MCF-7、 SKBR-3,MCF-10A;BALB/c nude	TIMP1↓	Neutrophil Extracellular Traps↓ Formationlung metastasis ↓	[164]
23	Phenylpropanoids	Senkyunolide H		*Angelica sinensis* (Oliv.) diels	Py230、C57BL/6 J	CXCR2↓	CD8^+^T↑	[165]
24	Quinones	Cryptotanshinone		Salvia *miltiorrhiza* Bunge	MDA-MB-231、RAW 264.7、BMDMs;NOD CB17-Prkdcscid/NcrCrl、C57BL/6	IL-6、TNF-α、IL-12↑IL-10↓CD86、CD80、iNOS↑CD206↓M2: ASK1↑JNK/p38↑M1: ASK1↓	M2↓ M1↑	[166]
25	Flavonoids	Smilax glabra Roxb–Ethy	N/A	*Smilax glabra* Roxb	MMTV-PyMT mice、C57BL/6J	STAT3/HIF-1α↓	M1↑M2↓T cells↑CD4⁺ T↑CD8⁺ T↑	[167]
26	Polyphenols	Caffeic acid		*Lonicera japonica* Thunb.,*Artemisia capillaris* Thunb.,*Cynara scolymus* L.	mouse splenocytes, BALB/c(4T1)	N/A	M1↑	[168]
27	Complex mixture	Taraxacum mongolicum extract	N/A	*Taraxacum mongolicum* Hand.-Mazz	MDA-MB-231、MDA-MB-468、U937	IL-10/STAT3/PD-L1↓M2↓M1↑	proliferation↓migration↓invasion↓anti-tumor immunity↑	[93]
28	Complex mixture	Huaier extractum	N/A	*Trametes robiniophila* Murr	4T1;C57BL/6、BALB/c	PI3K、Akt、p-Akt、JNK、p-JNK↑p-p38 MAPK↓IFN-γ↑IL-4↓	DCs ,CD4+T↑IL-1β,IL-12p70↑Th1↑	[168]
29	Complex mixture	Baoyuan Jiedu decoction	N/A	Baoyuan Jiedu decoction	4T1;BALB/c	TGF-β/CCL9↓	MDSCs↓	[169]
30	others	Cordycepin		*Cordyceps militaris* (L.) Fr	MCF7、 MDA-MB-231; human monocyte cell line (THP-1)	ALB↑CD86↑NOS2↑CD206↓	anti-tumor immunity↑	[170]
						ARG1↓IL6↑TNF↑		
						B cells↑CD8⁺ T↑		
31	Terpenoids	a4		*Salvia miltiorrhiza* Bunge	MDA-MB-231	PI3K/Akt/mTOR↓	PD-L1↓	[171]
32	Flavonoids	Quercetin		*Vaccinium * *corymbosum*,*Brassica oleracea* var.italica,*Allium cepa,Camellia sinensis*	RAW264.7	SIRT1/PGC-1α↑	Reprograms Immunometabolism of Macrophages	[172]
33	Flavonoids	Luteolin		*Daucus carota* L.,*Brassica oleracea* var. italica,*Perilla frutescens*(L.) Britton,*Apium graveolens* L.	BT-20、BT-549	AKT/mTOR↓	MMP9 ↓	[173]
34	Flavonoids	Quercetin		N/A	MCF-7; BALB/c nude	targeting calcineurin,calcineurin↓VEGF↓VEGFR2 ↓NFATc3↓	angiogenesis↓	[174]
35	Flavonoids	EGCG		*Camellia sinensis* (L.) Kuntze	MDA-MB-231、MDA-MB-468、BT-20、HCC-70、MDA-MB-157	PPARγ↓C/EBPα↓p-STAT3 ↓Altered the secretome profile of mature adipocytes	mature adipocytes↓the pro-migratory and pro-vasculogenic mimicry effects ↓	[175]
36	Flavonoids			*Camellia sinensis* (L.) Kuntze	4T1;Balb/c	HIF1α↓ GLUT1↓glucose↓lactic acid↓ VEGF↓ HK, PFK, LDH↓	glucose uptake,lactate production, ATP↓	[176, 177]
37	Terpenoids	Ursolic Acid		N/A	MCF-7,MDA-MB-231	AGO2↓PTEN↑	EMT↓CSCs↓	[178]
						FAK/PI3K/Akt/mTOR↓		
						miR-9↓miR-221↓slug↓vimentin↓		
						Nanog↓ Oct4↓c-Myc↓		
38	Terpenoids	AGS-30		*Andrographis paniculata* (Burm. f.) Nees	4T1,HUVECs,RAW264.7,THP-1;BALB/c	M2↓	metastasis↓	[179]
39	Terpenoids	Andrographolide		*Andrographis paniculata* (Burm. f.) Nees	MDA-MB--468,MDA-MB-231,E0771;4T1-implanted orthotopic mouse,BALB/c,C57BL/6	NF-κB-thoc1↓	CSCs↓	[180]
40					MCF-7,MDA-MB-231,T47D,MDA-MB-361,BT549,HUVECs,BALB/c nude	p300 ↓VEGF↓	COX-2↓ angiogenesis↓	[181]
41	Terpenoids	Celastrol		*Tripterygium wilfordii* Hook.b f	MDA-MB-231,HUVECs,BALB/c nude	DEGS1、CD146、S1PR1-3↓S1P↓	angiogenesis↓	[182]
42	Terpenoids	Asiatic acid		*Centella asiatica* (L.) Urb	4T1,HUVECs,BALB/c	VEGF/VEGFR2↓	angiogenesis and blood vessel permeability↓	[183]
43	Terpenoids	Fucoxanthin		*such as Undaria pinnatifida* (Harv.) Suringar, *Petalonia binghamiae*,*Sargassum siliquastrum,Hijikia* *fusiformis*	MDA-MB-231	VEGF-A、VEGF-C↓	angiogenesis↓	[184]
44	Flavonoids	Jaceidin		*Chiliadenus montanus* (Vahl) Brullo	MCF-7;Ehrlich's ascites carcinoma	VEGF-B/VEGFR-2↓	angiogenic↓	[185]
45	Flavonoids	Astragalin		*Astragalus membranaceus* (Fisch.) BungePersimmon, *Morus alba* L.,*Cuscuta chinensis Lam*	MDA-MB-231;HUVECs, CAM	Targets AKT、ZEB1、VEGFand MMP-9	angiogenic↓	[186]
46	Flavonoids mixture	Anthocyanins		*Prunus avium* (L.) L	4T1; BALB/C	Akt/mTOR↓ ERK1/2↓	angiogenesis↓	[187]
47	Polyphenols	Pterostilbene		*Vaccinium* spp	EMT6、4T1;BALB/c	caspase-3/Gasdermin E signaling pathway↑	Th,CTL,NK cells,B cells↑Treg,MDSC↓M2↓M1↑	[188]
48	Polyphenols	Matairesinol		N/A	MDA-MB-231,THP-1	Targeting M2	M1↑M2↓	[96]
49	Polysaccharides	Crude polysaccharide extract	N/A	*Auricularia polytricha* (Mont.) Sacc	THP-1,MCF-7、MDA-MB-231	NF-κB pathway↑	M1↑M2↓	[94]
50	Terpenoids	Glycyrrhetinic acid		*Glycyrrhiza uralensis* Fisch	4T1,RAW 264.7,THP-1,MDA-MB-231,HUVECs;BALB/c	JNK1/2↑	M1↑M2↓VEGF、MMP9、MMP2、IL-10↓ CD206、Arg-1↓sangiogenesis、 lung metastasis↓	[97]
51	Complex mixture	*Smilax china* L. Rhizome extract	N/A	*Smilax china* L	4T1,C57BL/6,MMTV-PyMT,BALB/c	ERK1/2 phosphorylation↓	M1↑M2↓CD8 + T↑	[98]
52	Polysaccharides	Lycium barbarum polysaccharide(50-100kDa)	N/A	*Lycium barbarum* L	4T1-Luc,BMDMs、BALB/c、C57BL/6	TLR4/NF-κB↑	M2↓M1↑	[99]
53	Polysaccharides	Grifola frondosa polysaccharide	N/A	*Grifola frondosa* (Dicks.) Gray	4T1;BALB/c	TIGIT↓MDSCs↓	immune responses↑	[103]
						Granzyme B↑CD8⁺ T↑		
54	Coumarins	Wedelolactone		*Eclipta prostrata* (L.) L	BMDMs,4T1 orthotopic murine breast cancer,BALB/c	TGF-β1/Smad↓	metastasis ↓	[190]
55	Complex mixture	Aurantiamide acetate,benzyl isothiocyanate		*Moringa oleifera* Lam	N/A (In silico only)	HIF-1α/VEGF/GLUT1↓	a potential natural inhibitor of the HIF-1α/VEGF/GLUT1 axi	[134]
56	Alkaloids	Sanguinarine		*Sanguinaria canadensis* L，*Chelidonium majus* L., *Argmone mexicana* L.	T47D,MDA-MB-231;BALB/c nude	hypoxia-induced activation of EphB4 and HIF-1α pathways↓	hypoxia-induced pathways↓	[135]
57	Quinones	Plumbagin		*Plumbago indica* L.	MCF-7	HIF-1α↓VEGF-A↓ VEGFR-2↓independent of PI3K/Akt/mTOR pathway	may have anti-angiogenesiseffect	[137]
58	Alkaloids	Cryptolepine		*Cryptolepis sanguinolenta* (Lindl.) Schltr,*Cryptolepis triangularis*	T47D、MCF-7、 MDA-MB-231、4T1;BALB/c	MAPK and mTOR signaling pathways↓	blocked HIF-1-mediated aerobic glycolysis (the Warburg effect)	[139]
						p-eIF4E↓HIF-1α↓		
59	Benzoquinones	Coenzyme Q 0		*Antrodia cinnamomea* (T.T. Chang & W.N. Chou) Sheng H. Wu, Ryvarden & T.T. Chang	MDA-MB-231、MDA-MB-468	HIF-1α↓	NLRP3-mediated inflammation, EMT/metastasis,The Warburg effects ↓	[140]
60	Terpenoids	Cryptanshinone		*Salvia miltiorrhiza* Bunge	MCF-7、MDA-MB-231	PKM2/β-catenin↓	Glycolysis↓ Cell Migration↓	[141]
61	Complex mixture	Compound kushen injection	N/A	*Sophora flavescens* Ait,*Heterosmilax japonica* Kunth	MDA-MB-231、MDA-BoM-1833;BALB/c nude	HIF-1α/SREBP1↓ PPARα/SLC47A1↓	PUFAs↑ ROS↑	[144]
62	Complex mixture	Hirsutella sinensis fungus	N/A	*Cordyceps sinensis* (Berk.) Sacc	MMTV-PyMT, BALB/c(4T1-Luc)	T-bet↓Eomes↑	lung metastasis↓ proportions of effector/memory T cells↑ proportions of exhausted T cells (Tex),Tregs↓ CD8 + T↑	[188]
63	Polyphenols	Curcumin		*Curcuma longa* L	CAFs and PBMCs	COX-2↓PGE2↓T-bet↑	Th1↑	[189]
						COX-2↓PGE2↓TGF-β↓ T-bet↑IFN-γ↑FoxP3↓		

### Targeting amino acid metabolism: reshaping the metabolic-immune nexus

**Fig. 2:**
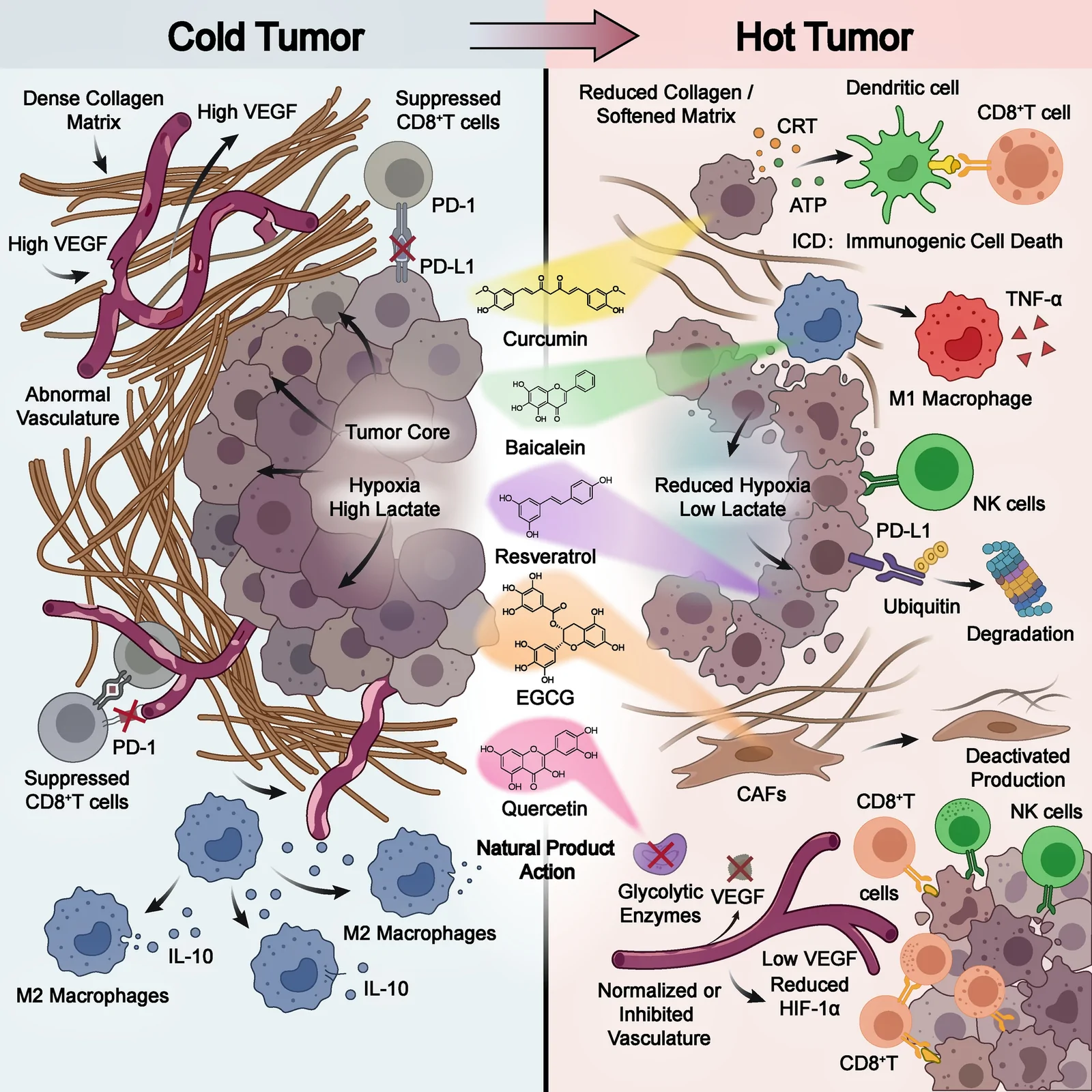
Mechanisms of natural products targeting the breast cancer TME

Amino acid metabolism is increasingly recognized as an important component of metabolic reprogramming in breast cancer, not only because it supports tumor biosynthesis and redox balance but also because it shapes immune-cell fitness within the TME [69, 192]. Rapidly proliferating tumor cells compete with infiltrating immune cells for key amino acids, thereby creating a nutrient-deprived and immunosuppressive niche [193]. In this context, NPs may help disrupt tumor metabolic dependence while partially restoring conditions favorable for anti-tumor immunity [194, 195]. One representative target is glutamine metabolism: breast cancer cells often upregulate glutamine transporters and glutaminase 1 (GLS1), which supports tumor growth while limiting nutrient availability for CD8 + T cells [196–198]. Natural products such as Momordin Ic [199], and Ginsenoside CK [200] have been reported to inhibit GLS1 directly or suppress the PI3K/AKT/mTOR-MYC axis, thereby reducing tumor glutamine consumption. In parallel, dysregulated tryptophan and arginine metabolism also contributes to immune escape. IDO1-mediated tryptophan catabolism generates kynurenine, which promotes regulatory T-cell expansion and impairs effector T-cell function [201, 202]. whereas Arg-1 derived from MDSCs and M2-like macrophages depletes L-arginine and induces T-cell hyporesponsiveness [203–205]. Compounds such as EGCG [177], Hypericum alpestre extract [206], Delicaflavone [207], and Yiai Fuzheng decoction [208, 209], have shown the capacity to interfere with these pathways and attenuate metabolic immunosuppression. In addition, some NPs may target serine and one-carbon metabolism, further exposing metabolic vulnerabilities in metastatic breast cancer cells [210]. Collectively, these findings suggest that NP-mediated regulation of amino acid metabolism may help relieve nutrient competition and reduce the accumulation of immunosuppressive metabolites, thereby contributing to metabolic and immune normalization of the TME (Fig. 2).

## Representative natural products and their mechanisms of action

Natural products are diverse, yet polyphenols, alkaloids, and saponins have demonstrated the most significant potential and possess the deepest research foundation in regulating the breast cancer tumor microenvironment. This chapter focuses on several highly representative natural products, elucidating their unique anti-cancer mechanisms and prospects for clinical translation.

### Polyphenols

Polyphenols are the most widely distributed secondary metabolites in the plant kingdom and have garnered significant attention for their remarkable antioxidant, anti-inflammatory, and immunomodulatory activities.

#### Curcumin

As a key regulator of the TME, Curcumin's mechanisms of action are multifaceted, covering the regulation of immune cell functions, phenotypic remodeling of stromal cells, blockade of signaling pathways, and modification of the physical microenvironment [211, 212].

regarding immune regulation, Curcumin can reverse the immunosuppressive state. A key role is restoring the cellular function of NK cells. It effectively reverses immune suppression mediated by breast cancer exosomes by acting on the key Jak3/STAT5 signaling pathway within NK cells, enabling them to regain their tumor-killing capacity [211]. Furthermore, Curcumin can polarize pro-tumor M2 macrophages into the anti-tumor M1 phenotype. The core mechanism lies in inhibiting the STAT3 signaling pathway, a key switch that maintains the M2 phenotype [213]. This mechanism is relevant to clinical drug resistance.

In stromal cells, Curcumin possesses important regulatory functions. It can block the transformation of adipose-derived mesenchymal stem cells into CAFs by inhibiting the C-X-C motif chemokine ligand 12(CXCL12)/C-X-C chemokine receptor 4 (CXCR4) axis, thereby breaking the malignant positive feedback loop between them [214], Moreover, it can directly "reprogram" existing CAFs. By downregulating the expression of cyclooxygenase-2(COX-2) and alpha-smooth muscle actin(α-SMA) in CAFs, Curcumin significantly reduces the secretion of immunosuppressive molecules such as prostaglandin E2(PGE2) and TGF-β,creating favorable conditions for restoring the anti-tumor immune response [189].

Beyond the direct regulation of cellular components, Curcumin can profoundly alter the physical and biochemical characteristics of the TME, particularly by mitigating tumor hypoxia, a core driver of malignancy. It does not directly attack HIF-1α,which accumulates massively during hypoxia, but rather induces the degradation of its binding partner, the ARNT (HIF-1β) protein, fundamentally weakening the tumor's adaptive capacity to hypoxia [215]. This effect is particularly evident in breast cancer stem-like cells (CS-LCs), which are the root cause of recurrence, and induces a higher rate of apoptosis under hypoxic conditions [215]. Effective inhibition of the HIF pathway subsequently blocks downstream angiogenesis, invasion, and metastasis programs [212]. In the bone metastasis microenvironment, its oxidative metabolites can inhibit the TGF-β/Smad "vicious cycle" [216]; while on a physical level, it can directly hinder cancer cell invasion by inhibiting the TEAD4-fibronectin adhesion axis [217].

In summary, Curcumin provides a strong theoretical basis for TME-targeted therapy in breast cancer through immune suppression reversal, matrix remodeling, reduction of hypoxic adaptation, and inhibition of cell invasion.

#### Resveratrol

Resveratrol (RES), a stilbene compound widely found in grape skins, is a potent regulator of the TME. In terms of the immune microenvironment, RES enhances the immune clearance efficiency of natural killer (NK) cells against breast cancer cells. Recent studies indicate that RES downregulates miR-17-5p to relieve its inhibition of MINK1, thereby activating the MINK1/JNK/c-Jun axis and significantly upregulating the NKG2D ligand ULBP2. This makes breast cancer cells more susceptible to recognition and elimination by NK cells [218]. Meanwhile, RES can also promote T cell-mediated immune responses. Research has found that RES inhibits PD-1 expression in T cells within lung tissue and promotes the polarization of macrophages toward the anti-tumor M1 phenotype, thereby significantly inhibiting lung metastasis of TNBC [219].

In the context of fibroblast interactions, CAFs are considered core drivers supporting cancer cell growth and invasion. RES can significantly inhibit CAF-induced tumor-promoting signals, such as Versican(VCAN) expression, cell proliferation, and organoid growth [220]. In CAF-tumor organoid co-culture models, RES reversed the malignant phenotypes driven by CAFs, suggesting its potential value in disrupting tumor-stroma interactions.

Energy metabolism is another key dimension of the TME. Both breast cancer cells and CAFs rely on highly active glycolysis to adapt to hypoxic and high-pressure microenvironments. RES can block metabolic adaptability by inhibiting key glycolytic enzymes and lactate accumulation. Recently, a RES delivery system based on layered double hydroxide nanoparticles significantly enhanced this effect, evidenced by decreased ATP levels, inhibited glycolysis rates, and impeded tumor growth [221]. Additionally, RES can reverse TGF-β1-induced migration and metastatic potential via the SIRT3/AMPK/autophagy axis, further reinforcing its regulatory role in the metabolic-signaling interaction network [222].

Notably, TME regulation is dose-dependent. Low-dose RES may exhibit pro-tumor adaptability, while high-dose RES possesses distinct anti-tumor effects. Studies show that Metformin can inhibit the adverse effects of low-dose RES and enhance the redox capacity and anti-tumor effects of high-dose RES [223], providing new insights for clinical combination therapy.

In summary, RES demonstrates the ability to systematically remodel the breast cancer TME by synergistically regulating immunity, fibroblast networks, energy metabolism, and signaling pathways. These evidences support the potential value of RES as a TME-targeted adjuvant anti-cancer strategy and provide an important theoretical basis for directions such as combination therapies and nano-delivery.

#### Epigallocatechin gallate

Epigallocatechin Gallate (EGCG), As the most abundant catechin in green tea, EGCG exhibits prominent performance in inhibiting cancer stem cells and regulating metabolism. EGCG can inhibit the pro-tumor reprogramming of cells derived from adipose tissue. Secretions from TNBC can drive adipose-derived mesenchymal stem cells (ADMSCs) to transform into a Cancer-Associated Adipocyte -like pro-tumor phenotype, accompanied by the activation of inflammatory factors such as Interleukin‑6 (IL-6), Interleukin‑1 beta(IL-1β), CCL2, and C‑C motif chemokine ligand 5 (CCL5). However, EGCG can significantly inhibit this transformation process and block the Smad2 and NF-κB signaling pathways, thereby attenuating the tumor's ability to "educate" the adipose microenvironment [224].

EGCG can counteract the inflammation and senescence induced by TNBC exosomes in stromal cells. Exosomes derived from TNBC cells can induce significant inflammatory responses and cell senescence in ADMSCs, characterized by marked increases in markers including Cyclin-dependent kinase inhibitor 1 A (p21), β-galactosidase(β-gal), IL-1β, and IL-6, as well as the activation of signaling pathways like protein kinase B signaling pathway (AKT), glycogen synthase kinase-3 beta (GSK-3β, and checkpoint kinase 2(CHK-2). Notably, EGCG treatment can significantly weaken the capacity of these exosomes to induce inflammation and senescence, while simultaneously reducing the mitochondrial components they carry, thereby decreasing the transfer efficiency of pro-tumor signals [225].

EGCG can modulate immunity and the gut microenvironment via natural exosome-like nanocarriers. Chen et al. (2022) developed natural exosome-like nanovesicles rich in EGCG from tea flowers. They found that tea flower exosome-like nanovesicles (TFENs) can promote ROS amplification in tumor cells in vivo, induce tumor cell death, and significantly reduce the growth of primary breast tumors and lung metastases. More importantly, TFENs can significantly regulate the composition of gut microbiota by increasing the abundance of beneficial bacteria and reducing inflammation-related strains. This improves the TME via the "gut microbiota-immunity-tumor axis," reducing the accumulation of MDSCs and enhancing the functional infiltration of CD4 + and CD8 + T cells [226].

EGCG can enhance the deep tumor tissue penetration capability of nanomedicines. A major obstacle in the TME is the dense ECM and acidic environment of solid tumors. In a phosphatase and acidity dual-responsive nanosystem designed by Zhou et al. in 2025, the introduction of EGCG achieved rapid activation and deep diffusion within the acidic tumor environment. EGCG within the system is capable of inducing a localized ROS amplification effect inside the tumor, thereby synergizing with chemotherapeutic drugs to successfully break through the dense ECM barrier, allowing effective drug retention and killing deep within the tumor [227].

#### Quercetin

The immunomodulatory effects of Quercetin have been extensively documented. In vivo studies demonstrate that Quercetin promotes the infiltration of CD4 + and CD8 + T cells within the tumor and enhances the secretion of Th1-type cytokines (IL-2, IFN-γ, TNF-α), thereby elevating cytotoxic immune activity. Concurrently, it reduces the levels of Th2-type factors (IL-4, IL-5, IL-13) and the proportion of Tregs, reversing the immunosuppressive cellular environment [228]. Additionally, Quercetin promotes the differentiation of γδ T cells into the anti-tumor Vδ2 subpopulation and enhances their cytotoxicity against breast precancerous lesions and solid tumor cells [120]. At the macrophage level, Quercetin inhibits M2-TAM and drives their polarization toward the M1 phenotype. This reduces the expression of immunosuppressive factors such as IL-10 and TGF-β, helping to restore the anti-tumor immune circuit.

Secondly, Quercetin influences the inflammatory and immune escape phenotypes of the TME by regulating multiple key signaling pathways. It inhibits the COX-2/PGE₂ axis incancer and immune cells, attenuating the PGE₂-mediated inhibition of DC maturation, NK cell recruitment, and T cell activity, thereby improving the immune activation threshold [229]. Furthermore, by regulating the JAK/STAT1 pathway, Quercetin upregulates interferon-γ receptor(IFNγ-R) and p-STAT1 while downregulating PD-L1 expression, increasing the sensitivity of tumor cells to T cell-mediated killing [120]. In some studies, Quercetin is also negatively associated with TGF-β signaling, thereby inhibiting EMT and fibrotic responses, and alleviating the matrix-related immune-excluded TME [230].

Metabolic remodeling is another key dimension of Quercetin's regulation of the TME. Quercetin significantly reduces lactate production in breast cancer cells by inhibiting Akt-mTOR signaling to induce autophagy and downregulating key glycolytic enzymes such as GLUT1, LDHA, and PKM2 [231]. Lactate accumulation is a core driver of the immunosuppressive TME; its reduction helps improve the pH gradient and enhance T cell and NK cell function. Additionally, Quercetin inhibits CPT1A-mediated fatty acid oxidation, blocking energy adaptive mechanisms in metastatic tumors [232], and weakening immune escape and drug resistance at the metabolic level.

**Fig. 3:**
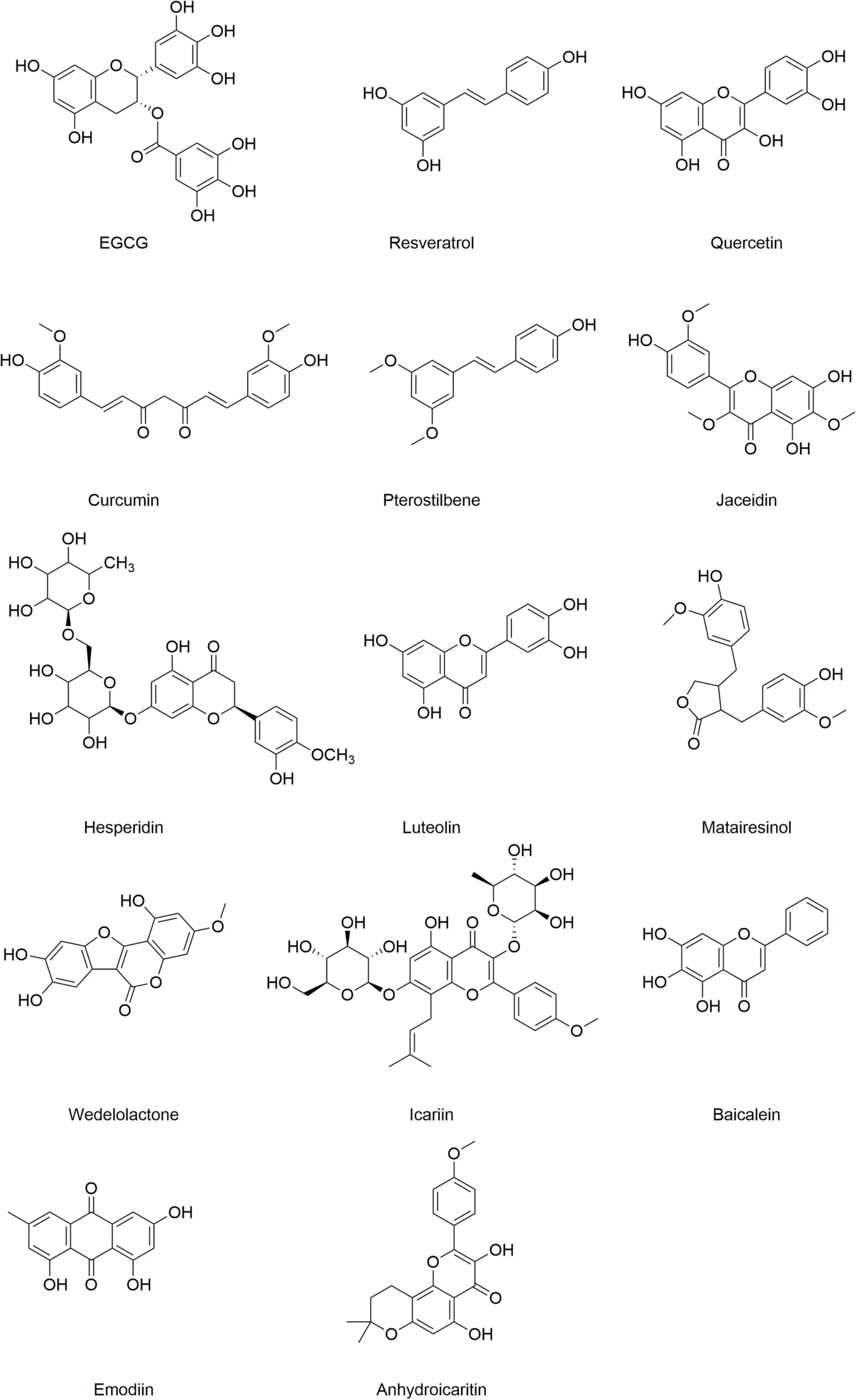
Chemical structures of polyphenols affecting the TMEPlease replace Figure 3 with the updated version provided in the attachment, named "figure3.tif".In this revised figure, the following changes have been made:The incorrect chemical structure of Hesperidin has been corrected.Emodin, Icariin, Baicalein, Wedelolactone, and Anhydroicaritin have been added (these compounds were previously misclassified as terpenes and have now been correctly relocated to Figure 3).View

Finally, Quercetin can also improve the tumor stromal barrier. By reducing Wnt16, α-SMA, and collagen deposition, it attenuates the activity of tumor-associated fibroblasts and improves the permeability of nanomedicines and immune cells within the tumor [233] (Fig. 3).

In summary, through the multi-dimensional synergistic effects of immune remodeling, metabolic regulation, and matrix modulation, Quercetin significantly improves the breast cancer TME. This provides a strong biological basis for targeting the immunosuppressive microenvironment and enhancing sensitivity to combination therapies.

### Alkaloids

Alkaloids are a class of nitrogen-containing organic compounds often possessing significant cytotoxicity and target specificity.

#### Berberine

Berberine (BBR),is an isoquinoline alkaloid, BBR has demonstrated potential as a pleiotropic regulator of the breast cancer TME. Its therapeutic effects are not limited to direct cytotoxicity but extend to the coordination of systemic anti-tumor immunity and the normalization of the microenvironmental niche. Remodeling the immunosuppressive landscape is one of its key mechanisms: Berberine-based nanoregulators have been confirmed to downregulate key immune checkpoints, including PD-L1 and IDO-1, thereby enhancing responses to photothermal therapy and chemo-immunotherapy in TNBC [234, 235]. This immune restoration is further amplified by BBR-induced immunogenic cell death. Recent evidence suggests that BBR acts as a potent GSDME activator to induce pyroptosis, subsequently triggering local and systemic immune surveillance to block tumor metastasis [236]. Additionally, combination strategies (such as Berberine combined with exercise) have been reported to significantly increase NK cell infiltration and recalibrate cytokine profiles in the TME of 4T1 mouse models [237].

Beyond immune activation, Berberine targets the hypoxic and inflammatory foundations of the TME. It mitigates hypoxia-driven tumor progression and reduces the loss of E-cadherin by inhibiting HIF-1α and modulating the gut microbiota-metabolite axis [152]. This aligns with observations of BBR disrupting the inflammatory feedback loops that maintain breast cancer stem cells; specifically, BBR inhibits BCSC expansion by regulating miRNA networks and the IL-6 signaling pathway [234, 238]. At molecular and epigenetic levels, BBR exerts inhibitory control over cell plasticity and drug resistance. It not only effectively reverses EMT and inhibits the SIRT1/AKT signaling pathway [239], but also intervenes in RNA epigenetics by targeting METTL3-mediated m6A modification of FGF7 mRNA [240].

**Fig. 4:**
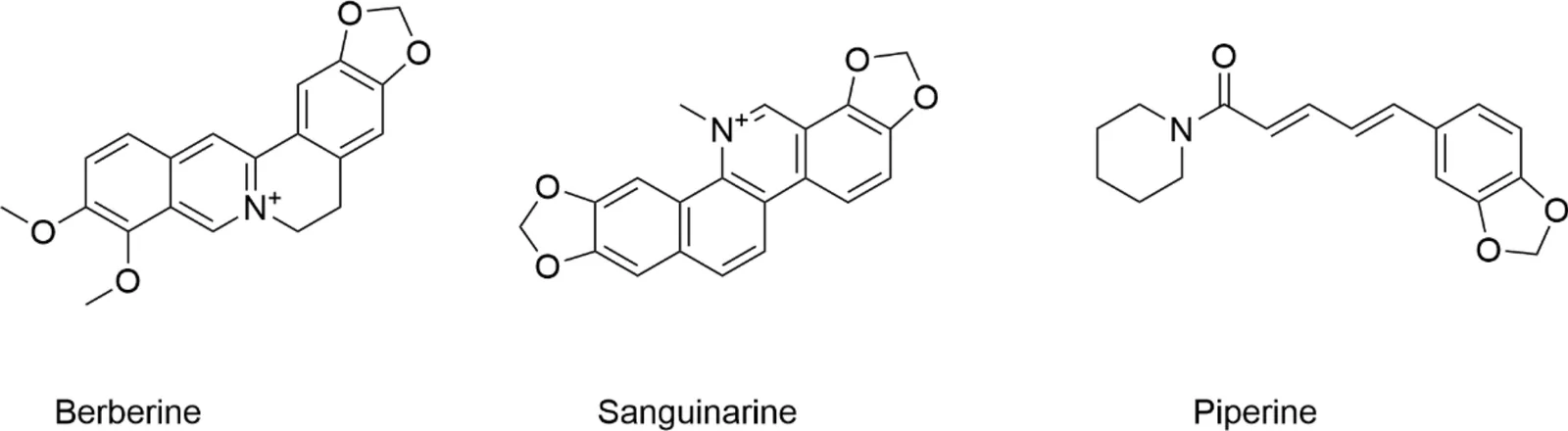
Chemical structures of alkaloids affecting the TME

In summary, these multi-level regulations spanning immune checkpoint blockade, hypoxic adaptation, and epigenetic reprogramming constitute the basis for Berberine to reverse multidrug resistance (MDR) and dismantle the pro-tumor microenvironment [241]. It is capable of orchestrating the reversal of pro-tumor inflammatory and hypoxic cascades. Its core mechanism involves disrupting the inflammatory cytokine loops that fuel metastasis. BBR can block the nuclear translocation of NF-κB p65 by preventing the degradation of IκBα, thereby abolishing LPS-induced inflammatory responses in MDA-MB-231 cells. This blockade effectively downregulates the secretion of key pro-inflammatory cytokines, including TNF-α and IL-6, which are crucial for maintaining the metastatic microenvironment [242] (Fig. 4).

### Saponins

#### Ginsenosides

As the primary active ingredients of Panax species, ginsenosides possess unique steroid structures and exhibit multi-dimensional regulatory capabilities in remodeling the breast cancer TME, particularly in reversing MDR. Unlike single-target drugs, ginsenosides systematically dismantle the tumor survival niche by synergistically regulating tumor stemness, metabolic reprogramming, and immune surveillance.

Targeting tumor stemness and reversing the roots of resistance eradicating CSCs is a key strategy for overcoming breast cancer recurrence and chemotherapy resistance. Ginsenoside Rg3 exhibits significant anti-stemness activity. It not only inhibits CSC self-renewal by activating the Hippo signaling pathway [243], but also accelerates the degradation of the oncogene MYC mRNA by upregulating the let-7 microRNA family, thereby blocking stemness maintenance at the post-transcriptional level [244]. Furthermore, Rg3 reduces CD44 expression, consequently diminishing the clonogenic potential of CSCs [20]. In the context of paclitaxel resistance, Ginsenoside Panaxatriol resensitizes TNBC cells by repressing stemness-associated genes (OCT4, SOX2, NANOG) and blocking the IRAK1/NF-κB and ERK pathways, which subsequently reduces the secretion of pro-survival inflammatory cytokines (IL-6, IL-8, CCL2) [245].

Metabolic reprogramming and oxidative stress targeting the metabolic vulnerability of TNBC is another key strategy. Ginsenoside Compound K disrupts glutamine metabolism by downregulating glutaminase 1. This inhibition limits glutamate production and amino acid utilization, leading to ATP depletion and severe ROS accumulation that triggers apoptosis [200]. Similarly, the novel derivative 2-Deoxy-Rh2 exerts dual-targeting effects by dampening glycolysis and inhibiting mitochondrial respiration, effectively starving the tumor cells [246].

**Fig. 5:**
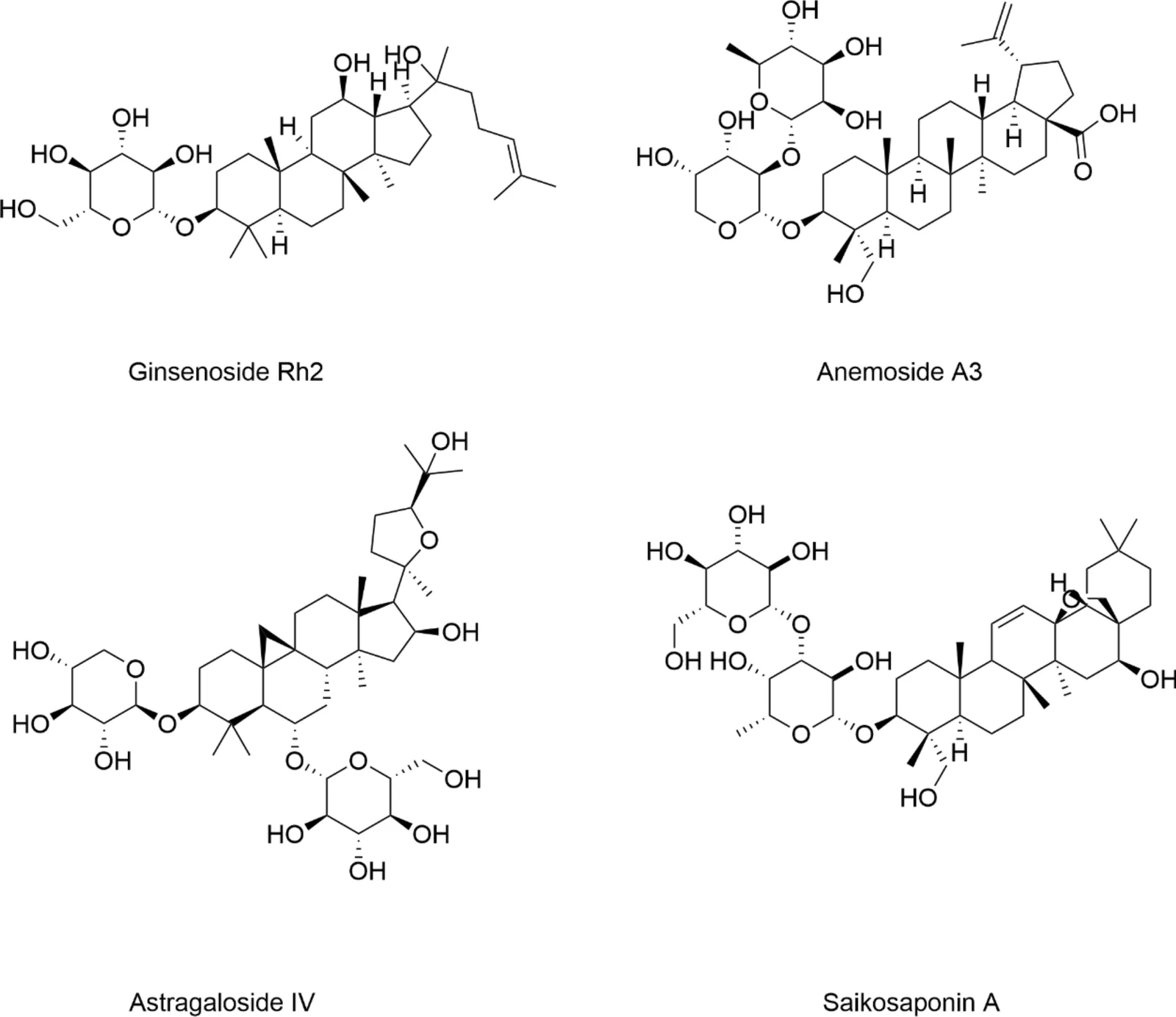
Chemical structures of saponins affecting the TME

Immunomodulation and Metastasis Inhibition Ginsenosides significantly enhance the visibility of tumors to the immune system. A study demonstrated that Ginsenoside Rh2 enhances NK cell surveillance by inhibiting Endoplasmic Reticulum protein 5 in breast cancer cells [156]. Additionally, Rh2 regulates TNBC proliferation via the IL-6/JAK2/STAT3 signaling axis [247]. To curb metastasis, Ginsenoside Rh1 inhibits the migration of MDA-MB-231 cells by suppressing MMP-2, MMP-9, and VEGF-A expression at both mRNA and protein levels [248], while Ginsenoside Rg1 downregulates EMT-associated markers to prevent invasion [249] (Fig. 5).

**Fig. 6:**
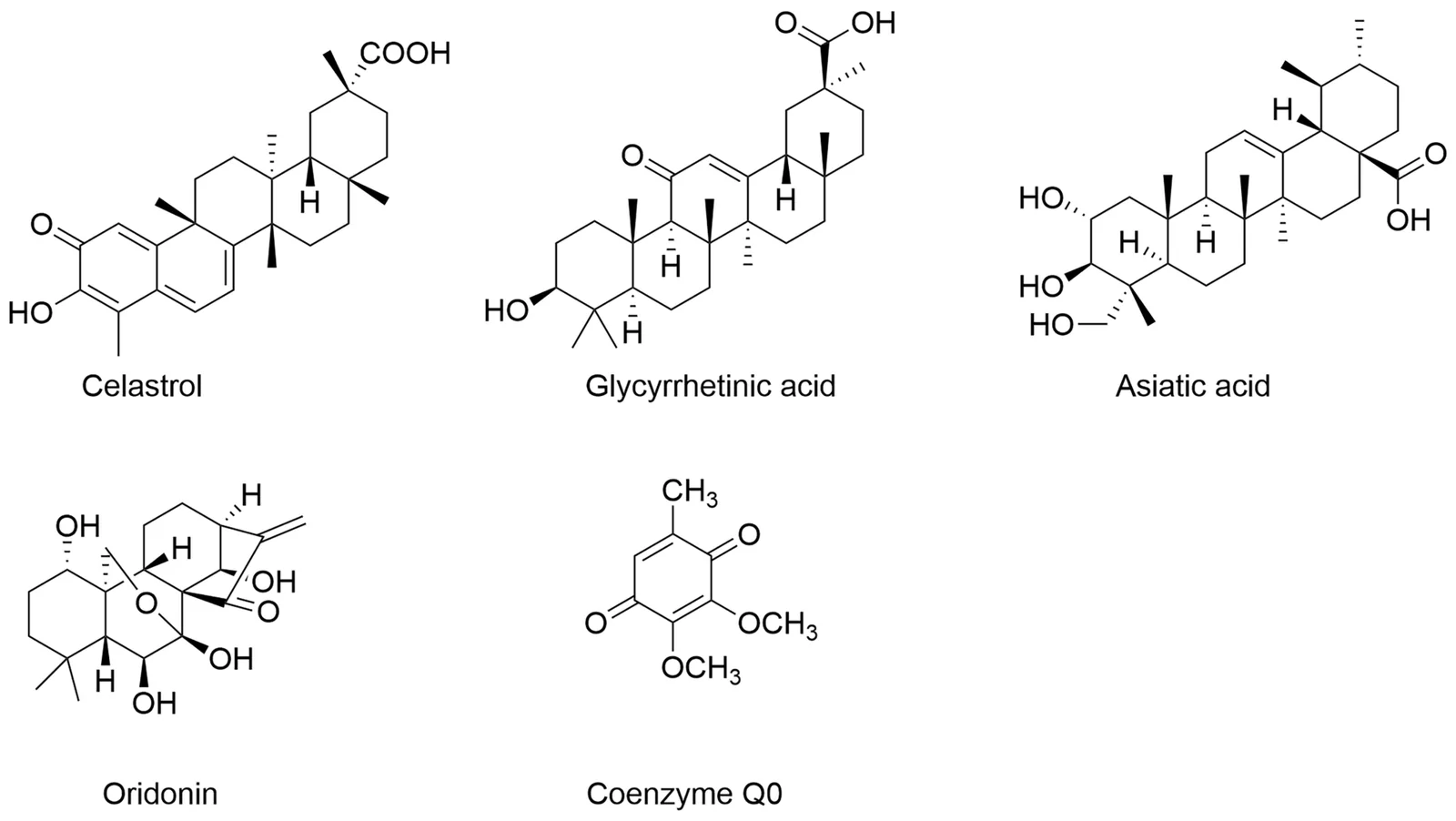
Chemical structures of Non-saponin terpenoids and other compounds affecting the TME

Nanotechnology and Safety Profile Recent advances in nanotechnology have further optimized the therapeutic index of ginsenosides. Long et al. (2024) developed a Ginsenoside Rh2-irrigated nano-in-thermogel system, which effectively combated multidrug resistance by ensuring sustained local drug delivery [250]. Beyond efficacy, Rh2 also demonstrates a protective role against chemotherapy-induced toxicity; it mitigates doxorubicin-induced cardiotoxicity by inhibiting cardiomyocyte apoptosis and inflammatory damage, highlighting its potential as an adjuvant agent to improve the safety of standard chemotherapy regimens [251] (Fig. 6).

### Marine natural products

Marine-derived secondary metabolites are emerging as promising anticancer agents due to their structural diversity and potent bioactivity [252].

#### Fucoxanthin

Fucoxanthin (FX), a marine carotenoid mainly isolated from brown algae, has attracted attention for its potential to interfere with vascular remodeling and metastatic progression in breast cancer. In TNBC-related models, FX has been reported to inhibit tumor cell proliferation and induce apoptosis while suppressing angiogenesis and lymphangiogenesis through downregulation of VEGF-A, VEGF-C, and VEGFR-3 expression [184, 253]. These effects suggest that FX may limit both local vascular support and routes of metastatic dissemination.In addition to its anti-angiogenic activity, FX has been shown to interfere with later steps of the metastatic cascade. FX can reduce the expression of endothelial adhesion-related molecules, weaken the interaction between circulating tumor cells (CTCs) and the vascular endothelium, and inhibit transendothelial migration [254, 255].Through these mechanisms, FX may help suppress distant metastatic colonization.

Recent nanotechnology-based studies further suggest that the TME-modulating effects of FX can be strengthened through combination delivery strategies. For example, hydroxyethyl starch-cholesterol self-assembled nanoparticles co-delivering FX and Twist siRNA have been used to simultaneously target EMT-related transcriptional programs and tumor invasiveness [256]. In this system, Twist silencing cooperated with FX to increase epithelial markers such as E-cadherin while decreasing mesenchymal markers, thereby attenuating the invasive phenotype of TNBC cells [256, 257].

#### Fucoidan

Fucoidan is a sulfated polysaccharide derived from brown algae that has shown multiple TME-related effects in breast cancer. Under hypoxic conditions, fucoidan has been reported to suppress HIF-1α-related signaling and inhibit EMT-associated transcriptional programs, thereby reducing invasive behavior [258]. In addition, fucoidan-based nanoplatforms have been engineered to deliver oxygen or respond to tumor-associated stimuli, which may help alleviate local hypoxia and improve the efficacy of photodynamic therapy [259]. Fucoidan has also been associated with reduced VEGF expression and decreased microvessel density, supporting its anti-angiogenic potential [260]. Notably, oligo-fucoidan has been reported to enhance the anti-tumor activity of olaparib by aggravating homologous recombination deficiency and increasing DNA damage in TNBC models [252].

**Fig. 7:**
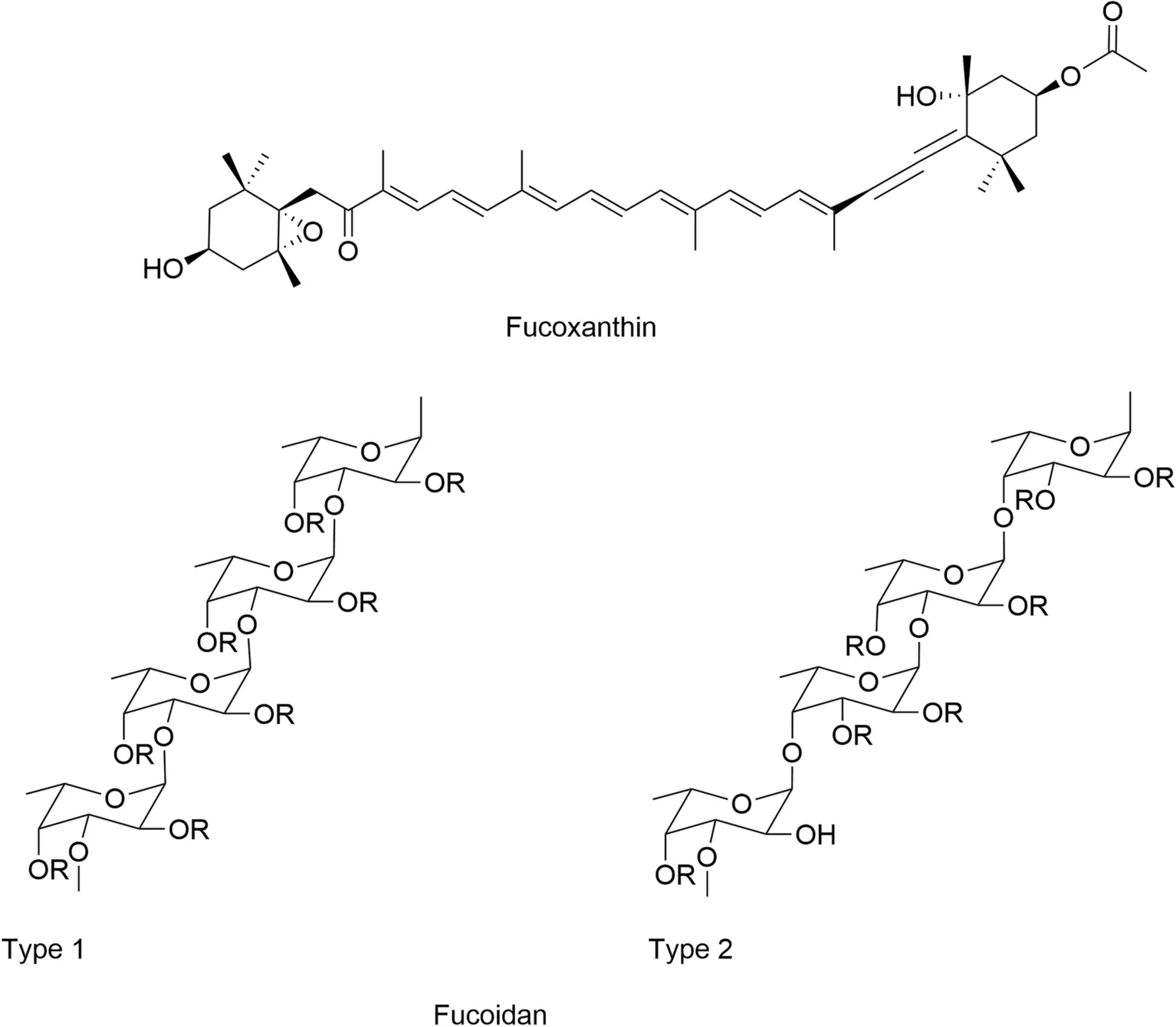
Chemical structures of marine natural compounds affecting the TME Fucoidan:Classical main chain structure of fucoidan type 1 chain composed of α（1→3）linked;type 2 chain composed of altering α（1→3）and α（1→4）linked fucose.R=H,fucose,xylose,galactose,mannose,glucuronic acid…or SO_3_-.

Beyond hypoxia and angiogenesis, fucoidan may also contribute to immune remodeling. Fucoidan-containing nanoframeworks combined with photodynamic or thermal therapies have been shown to induce ICD, leading to the release of ATP, HMGB1, and tumor-associated antigens [259, 261]. These signals can promote dendritic-cell maturation and cross-presentation, thereby facilitating CD8 + T-cell infiltration and activation. Some studies further suggest that fucoidan-based systems may support sustained T-cell cytotoxicity in metabolically adverse tumor settings [262] (Fig. 7).

## Combination therapy strategies involving natural products

Monotherapy with natural products often shows limited efficacy in breast cancer because of marked intratumoral heterogeneity, adaptive resistance, and restricted drug exposure within the tumor site. Nevertheless, the multi-target nature and relatively favorable safety profiles of many NPs make them attractive candidates for combination therapy, particularly when the goal is to sensitize tumors through TME remodeling rather than to rely on direct cytotoxicity alone [263].

### Natural products in combination with immune checkpoint inhibitors

The efficacy of immune checkpoint blockade (ICB) in breast cancer is strongly influenced by the pre-existing immune status of the TME. Tumors with weak antigen presentation, poor T-cell infiltration, high levels of MDSCs or Tregs, dominant M2-like macrophage populations, and marked stromal exclusion generally respond poorly to PD-1/PD-L1 blockade [25, 75, 76, 264]. NPs may help overcome these limitations by increasing tumor immunogenicity and restoring effector immune function.

One important mechanism involves the induction of immunogenic cell death (ICD) and activation of innate immune sensing pathways such as cGAS-STING. These processes promote the release of ATP, HMGB1, and related danger-associated signals, thereby facilitating dendritic-cell activation and cross-priming of CD8 + T cells [82, 265]. For example, cucurbitacin B has been shown to covalently target MTCH2 and activate cGAS-STING signaling, resulting in enhanced tumor immunogenicity and improved response to anti-PD-1 therapy [266].

In addition, NPs may reverse ICB resistance by reprogramming suppressive immune-cell populations. Since MDSCs, Tregs, and M2-like macrophages dampen cytotoxic lymphocyte activity and maintain local tolerance, their depletion or functional reorientation is highly relevant to checkpoint sensitization [25, 75]. For instance, 3,3′-diindolylmethane (DIM) inhibits MDSCs through the miR-21/PTEN/PIAS3-STAT3 axis and is associated with increased CD4 + and CD8 + T-cell infiltration as well as enhanced IFN-γ production, ultimately improving anti-PD-1 efficacy [267]. Similarly, ketoglutaric acid has been shown in TNBC models to increase CCL5 and CXCL10 expression, promote dendritic-cell activation, reduce Treg abundance, and enhance anti-PD-L1 responsiveness [268].

Furthermore, NPs may cooperate with ICB by alleviating stromal and metabolic barriers that prevent lymphocyte infiltration and persistence. Dense ECM deposition, CAF activation, oxidative stress, and lactate accumulation all contribute to immune dysfunction and exclusion [66, 124, 269]. Nano-puerarin, for example, has been reported to inhibit TGF-β/SMAD and NOX4 signaling, reduce α-SMA expression and ECM deposition, decrease Tregs and MDSCs, and increase M1-like macrophages and CD8 + T cells, thereby improving anti-PD-L1 efficacy [270].

### Natural products in combination with chemotherapy

The benefits of combining NPs with chemotherapy extend beyond simple enhancement of tumor cell killing. In breast cancer, chemoresistance is frequently shaped by the TME, including poor intratumoral drug penetration, CAF-derived survival signals, macrophage-mediated protection, ECM-associated drug sequestration, and hypoxia-driven metabolic adaptation [66, 271, 272] (Tables 3, 4). By modulating these features, NPs may sensitize tumors to conventional cytotoxic agents.

**Table 3:** Natural products in combined interventions for modulating the TME

No.	Natural products	Combined intervention	Cell lines or animal models	Mechanism of action	Result	Refs.
1	Astragaloside IV	Cisplatin	4T1、MDA-MB-231、MCF-7;BALB/c	p- STING↑	Boosted host defense inhibited tumor progression	[273]
				CD3⁺T↑CD11c⁺↑F4/80⁺↑		
2	Berberine	Exercise	4T1 ,MCF-7BALB/c	the infiltration of NK cells↑	immune system↑	[237]
3	Cordycepin	Doxorubicin	MDA-MB-231,BALB/c nude	TNF signaling pathway↑	MMP3↓MMP9↓Enhanced doxorubicin sensitivity and anti-tumor efficacy with low toxicity, CCL2↓ CXCL1↓	[271]
4	Curcumin	Quinacrine	HUVEC,BALB/cnude,Chicken embryo,MCF-10A	ABCG2↓VEGF A↓Ang-1↓	Angiogenesis,metastatic potential↓	[274]
				Ang-2↓PI3K-Akt-eNOS↓CD44↓CD133↓EMT↓		
5	Baicalin	5-Fluorouracil	Ehrlich Solid Tumor model in female Swiss albino mice	NF-κB signaling pathway↓	IL-1β↓VEGF↓	[275]
6	Piperine	Doxorubicin	MDA-MB-231;Ehrlich Ascites Carcinoma	PI3K/Akt/mTOR↓CSCs↓	Doxorubicin sensitivity↑ Inflammatory cells infiltration↓	[276]
7	3,3′-Diindolylmethane	Mouse PD-1 antibody	4T1、B16-F10; BALB/c (4T1); C57BL/6 J (B16-F10 melanoma)	miR-21↓ PTEN/PIAS3-STAT3↑	MDSCs↓CD4⁺T↑CD8⁺ T↑IFN-γ↑	[267]
8	Cucurbitacin B	Pembrolizumab	MCF-7MDA-MB-231,HCC1954,SK-Br-3,MDA-MB-453,HCC1937,BT-549,HCC1569,MCF-10A,4T1,MMTV-PyMT, BALB/c	Targeted MTCH2, cGAS-STING↑	PD-1 immunotherapy response↑	[266]
9	Celastrol liposomes	anti-PD-L1	4T1,3T3-L1,Balb/c	mPD-L1↓PD-L1⁺ EVs↓	T cell↑aPD-L1↑	[277]
					Remodeled the TME	
10	Ketoglutaric acid	α-PD-L1	4T1、MDA-MB-231、DC2.4;BALB/c	CD8 + T↑CCL5、CXCL10↑ DC↑Treg↓	IFN-γ↑Granzyme B↑Reprogramed the TME	[268]
				PD-L1↑(via STAT1↑		
11	Celastrol-loaded ginsenoside Rg3 liposomes	aPD-L1	MDA-MB-231,4T1,BALB/c	glucose-regulated protein 78↓ Endoplasmic reticulum↑	CD8 + T, dendritic cells↑immunosuppressive cells↓ T cells↑	[278]
12	Hesperidin PLGA nanoparticles	aPD-1	4T1,3T3-L1,BALBc	CCL2↓ ADPN↑M2, Tregs, MDSCs↓CD8 + T、M1、DCs↑	aPD-1↑	[279]
13	Nano-puerarin	α-PD-L1	4T1,NIH3T3,BPD6;Balb/c	ROS↓TGF-β/SMAD↓NOX4↓	CD8⁺ T↑Tregs, MDSCs↓M1↑	[270]
					α-SMA,ECM↓aPD-L1↑	
14	Resveratrol	Doxorubicin	MDA-MB-231,MCF-7/ADR,MCF-7，MCF-10A	SIRT1↑,β-catenin↓	E-cadherin↑ N-cadherin、Vimentin、β-catenin↓	[280]
15	Schisandrin C	cisplatin	4T1,BALB/c	cGAS-STING-type I↑	CD8⁺ T、NK cells↑	[91]
16	Curcumin	Paclitaxel	MCF-7,MDA-MB-231，THP-1	PI3K/AKT/STAT3↓	TGF-β↓M2↓	[95]
17	Baicalein	tamoxifen	MCF-7TR、T-47DTR, BT-474TR、ZR-75-1TR;NOD/SCID	HIF-1α↓	Aerobic glycolysis↓	[55]
					Reversed mitochondrial dysfunction	
18	Hesperidin	Doxorubicin	4T1，MCF-7,PBMCs，BMDSC,Balb/c	VEGF、MMP-2、MMP-9↓ IFN-γ↑IL-4↓	Metastasis↓	[281]
19	Ganoderma lucidum β-glucan	Gemcitabine	E0771, C57BL/6	Dectin-1 pathway	MDSCs↓M2↓Tregs↓ CD8⁺T↑Th1↑IFN-γ↑	[104]

**Table 4:** Natural products in different signal transduction pathways and their function

No.	Natural products	Combined clinical drugs	Nano-delivery system	Function	Refs.
1	Astragaloside IV	Oxymatrine	Co-loaded liposomes	Achieved tumor microenvironment-responsive drug release	[282]
				Enhanced the antitumor efficacy of α-PD-1 by boosting TIL activity and inhibiting CAF activation	
2	Emodin	N/A	Nanoparticles-loaded injectable hydrogel	facilitated enhanced penetration and uptake of EMO within the tumor microenvironment	[283]
3	Baicalein	N/A	mPEG-PLGA nanoparticle	TGF-βsignaling pathway↓,CAFs↓influenced the interstitial microenvironment,the infiltration of cytotoxic T cells↑	[284]
4	Berberine	doxorubicin	Encapsulate with hyaluronic acid and chitosan	CSCs↓PD-L1↓chemo-immunotherapy responsiveness↑	[234]
5	Berberine	Doxorubicin	pH-Sensitive chondroitin sulphate-based nanoparticles	anti-metastatic effect,fibroblasts↓,angiogenesis↓ extracellular matrix deposition↓	[285]
6	Puerarin	N/A	Nanoemulsion	Targeted tumor-associated fibroblasts(TAFs),TGF-β/SMAD and NOX4 signaling↓,α-SMA、ECM↓	[270]
				Improved immune microenvironment (IL-4, IL-6, IL-10, IL-13, CCL2, CCL5↓CD8⁺ T↑Tregs,MDSCs↓M1 ↑)	
7	Oridonin	N/A	PEG-PLGA nanoparticles	Inhibited angiogenesis and tumor metastasis	[286]
8	Berberine	Doxorubicin	Self-assembled nanodrug of doxorubicin and berberine, subsequently functionalized with 4T1 cell membranes coating	Pulmonary metastasis(via blocking HMGB1-TLR4 axis)↓	[287]
9	Berberine	N/A	Hyaluronic acid -coated berberine -indocyanine green self-assembly active nano modulator	PD-L1↓IDO-1↓	[235]
10	Silybin	Doxorubicin	Combined liposomal formulations	NF-κB activation in TAFs↓TAFs and fibrosis (α-SMA, collagen)↓ immunosuppressive cytokines (TGF-β、IL-6、SDF-1↓IFN-γ、IL-12↑) CD8⁺ T cells、DCs、NK cells↑Tregs、MDSCs↓	[288]
11	Naringenin	N/A	Nanoparticles	angiogenesis-related genes (Nestin, NRP-1, NRP-2, CD93)↓inflammation related genes (IL-1β, TNF-α, NF-κB)↓ angiogenesis↓	[289]
12	Curcumin	Celecoxib	HA-coated, TAT-NBD peptide-modified co-loaded liposomes (HA/TN-CCLP)	Synergistic inhibition of inflammatory signaling, responded to the TME by releasing drugs,intelligent cascade delivery & intracellular t	[290]
				Release, reprogramed the tumor inflammatory microenvironmen	
13	Curcumin	Doxorubicin	Liposomes of pegylated RGDK-lipopeptide	Tumor growth inhibition↑	[291]
14	Ginsenoside Rg3	Docetaxel	Rg3-based liposom	Chemosensitization and synergistic cytotoxicity	[292]
				TME Desmoplasia:TGF-β↓CAFs↓CD8 + ↑M1↑Treg, MDSC↓	
15	Tetrandrine	Docetaxel	Nanoparticle co-modified with anisamide and CAF cell membrane to load tetrandrine	CAFs↓collagen secretion↓Wnt/β-catenin pathway↓IGF2↓	[293]
16	Dihydroartemisinin	Redox paclitaxel prodrug	Nanoparticle	Released drug by cro-environmental triggering	[294]
17	Elemene	N/A	Nanoemulsion, Composed of soybean phospholipids, cholesterol, ethanol, and phosphate buffers	Scavenged reactive oxygen species in TME,HIF-1α、CD31↓, angiogenesis↓NLRP3 inflammasome↓IL-1β↓, pro-inflammatory signaling↓metastasis↓	[295]
18	*Plantago Asiatica* L. acidic polysaccharide	N/A	Dectin-1-targeted pH-responsive liposomal nanoplatform	p53 and NF-κB signaling pathways↑DCs↑ TAMs polarization, and cytotoxic T lymphocytes infiltration	[296]
19	Curcumin	Doxorubicin	Star polyester-based folate acid-targeting nanoparticles	Exhibited faster drug release in the acidic tumor microenvironment (pH 6.8) compared to physiological pH (7.4), enabling tumor-specific release and reducing systemic toxicity	[297]
				Reversal of multidrug resistance	
20	Andrographolide	Icariside II	Nanoparticles (andrographolide, Icariside II, and shRNA targeting UDP-N-acetylglucosamine 2-epimerase/N-acetylmannosamine kinase)	STAT3/TGF-β pathway↓, CD4 + 、CD8 + T↑M2↓M1↑	[298]

NPs can weaken stromal and physical barriers that limit drug delivery. Excessive ECM deposition and activated CAFs increase interstitial fluid pressure and restrict diffusion of chemotherapeutic agents into poorly perfused regions [66, 124]. Matrix remodeling by NPs may therefore improve intratumoral drug distribution [127, 128]. Additionally, NPs can interfere with paracrine survival signals generated by stromal and immune cells. M2-like TAMs, for example, promote chemoresistance by secreting TGF-β, IL-10, and other pro-survival mediators. Curcumin has been shown to suppress M2 macrophage-mediated paclitaxel resistance through inhibition of the PI3K/AKT/STAT3 pathway [95].

Natural products may also counteract hypoxia-related and metabolic forms of chemoresistance. HIF-1α signaling under hypoxia promotes tumor-cell survival, stemness, and anti-apoptotic programs, thereby reducing chemotherapy responsiveness [299, 300]. Baicalein has been reported to restore drug sensitivity in resistant models by suppressing HIF-1α, reducing aerobic glycolysis, and improving mitochondrial dysfunction [55]. Ginsenoside Rg3 similarly appears to reverse tamoxifen resistance by inhibiting glycolytic adaptation [301].

NPs may help eliminate TME-supported therapy-tolerant subpopulations, including cancer stem-like cells that persist in hypoxic, inflammatory, and fibrotic niches. Piperine, for example, enhances the sensitivity of TNBC cells to doxorubicin by inhibiting PI3K/Akt/mTOR signaling and reducing stemness-associated properties [276]. In some cases, NPs may also improve the therapeutic index of chemotherapy by reducing toxicity. Baicalin has been reported to strengthen the anti-tumor effect of 5-fluorouracil while alleviating treatment-associated toxicity through suppression of angiogenesis and inflammatory mediators such as VEGF and IL-1β [275].

### Natural products combined with anti-angiogenic or vascular-normalizing therapy

Angiogenesis is a fundamental pathological process driving the sustained proliferation and distant metastasis of breast cancer. Anti-angiogenic agents targeting the VEGF and its receptors have demonstrated distinct pharmacological efficacy in depriving tumors of their blood supply [302, 303]. However, anti-angiogenic monotherapy often induces adaptive responses within the TME, including compensatory neovascular programs and severe hypoxia, which may ultimately promote resistance and metastasis [304–306]. Excessive vascular pruning can impair oxygen delivery and drive chronic hypoxia in the TME [307]. In turn, this hypoxic state activates HIF-1α-dependent transcriptional programs that support vasculogenic mimicry (VM), epithelial-mesenchymal transition (EMT), and invasive behavior [308]. In TNBC models, anti-angiogenic therapy-induced hypoxia has been associated with enhanced metastatic dissemination despite partial suppression of primary tumor vascularization [309].

In this regard, NPs may serve as useful adjuvants because of their simultaneous effects on hypoxia, EMT, stemness, and stromal signaling. Oxymatrine provides a representative example. When combined with bevacizumab, oxymatrine has been shown to counteract bevacizumab-induced EMT and cancer stem cell-like traits under hypoxic and nutrient-stressed conditions by inhibiting Wnt/β-catenin signaling [310]. Through this mechanism, oxymatrine not only enhances the anti-tumor activity of bevacizumab but also reduces the risk of recurrence and metastasis by impairing stem-cell self-renewal [310].

At present, however, studies specifically examining NP combinations with anti-angiogenic or vascular-normalizing therapy remain limited. Given the central role of angiogenesis in breast cancer and the known microenvironmental liabilities of anti-vascular treatment, this area deserves more systematic investigation. Future work should focus not only on anti-vascular efficacy but also on whether NPs can preserve perfusion, reduce hypoxia, and improve drug and immune-cell accessibility within tumors.

### Nanotechnology-enabled co-delivery systems as platforms for synchronous tumor targeting and TME remodeling

Nanotechnology has substantially expanded the translational potential of NP-based combination therapies. Many NPs suffer from poor solubility, rapid metabolism, and limited tumor accumulation, all of which constrain their performance as free agents. Nanoscale delivery systems can help address these pharmacokinetic limitations while enabling coordinated release of NPs together with chemotherapeutic drugs, immunotherapeutics, gene modulators, or photothermal agents [127, 275]. Importantly, such systems can simultaneously target tumor cells and multiple TME compartments(Table.4).

Several studies illustrate this principle. A berberine-based nanoregulator was shown to downregulate PD-L1 and IDO-1 while integrating photothermal therapy, thereby enhancing chemo-immunotherapy responsiveness [235]. Dectin-1-targeted, pH-responsive liposomes carrying *Plantago asiatica* L. acidic polysaccharides promoted dendritic-cell activation, M1 macrophage polarization, and cytotoxic T-cell infiltration [296]. Likewise, nano-puerarin has been reported to improve checkpoint therapy while reducing fibrosis, oxidative stress, and immunosuppressive-cell infiltration [270].

## Current limitations and future perspectives

Although natural products have shown encouraging experimental results and immense therapeutic potential in regulating the breast cancer TME, the path of translational medicine from basic research to clinical application still faces numerous challenges. Objectively examining current limitations and planning future research directions based on these is crucial for promoting substantive progress in this field.

### Pharmacokinetic bottlenecks and standardization hurdles

The universally poor water solubility rapid metabolism, and low bioavailability of natural products remain the primary bottlenecks limiting their clinical efficacy [311]. For example, prominent molecules like curcumin and resveratrol undergo extremely rapid metabolism in vivo, making it difficult to reach effective therapeutic concentrations within tumor tissues. Although nanodelivery systems have alleviated this issue to some extent, challenges such as low drug loading, burst release effects, and the long-term safety of nanomaterials themselves require further evaluation. Furthermore, natural products possess complex sources with significant batch-to-batch variations. The lack of unified quality control standards and qualitative/quantitative systems for active ingredients challenges the reproducibility of experimental results and hinders regulatory approval as standardized drugs [312].

### Current strategies for NP standardization, quality control, and regulatory translation

Although lack of standardization remains a major barrier to the clinical translation of NPs, substantial progress has been made in improving formulation consistency, quality control, and regulatory feasibility. For complex NPs, particularly herbal extracts and botanical preparations, standardization requires an integrated framework that includes phytochemical profiling, marker-based quantification, batch-to-batch consistency assessment, source authentication, and contaminant control [313–316]. Modern analytical platforms such as HPLC, UHPLC-MS/MS, and NMR have become important tools for establishing chemical fingerprints and defining quality specifications [ 313, 317–319]. while chromatographic fingerprinting, chemometric analysis, and multi-marker strategies further enhance reproducibility, especially for multi-component products [314, 317, 320]. In addition, quality assurance is increasingly incorporating botanical authentication, DNA barcoding, stability testing, process control, and bioactivity-linked evaluation methods such as spectrum-effect relationship analysis [314, 315, 321–324]. From a regulatory perspective, these advances are gradually aligning with modern drug-development frameworks. Both the FDA botanical drug pathway and EMA/HMPC guidance emphasize raw-material control, chemistry-manufacturing-control (CMC), batch consistency, and rigorous safety and efficacy evaluation [315, 316, 325]. Together, these developments suggest that, although standardization remains challenging, it is becoming increasingly manageable and will be essential for the successful clinical translation of NP-based breast cancer therapies.

### TME heterogeneity and limitations in mechanistic research

Current mechanistic studies mostly focus on single targets or single cell types (e.g., focusing solely on fibroblasts or macrophages), neglecting the fact that the TME is a highly dynamic and complex ecosystem. Breast cancer, especially TNBC, exhibits high heterogeneity; TME characteristics,such as the degree of hypoxia and immune infiltration patterns, vary significantly between patients or even within different regions of the same tumor [326]. Current research often relies on homogenized cell lines or animal models, which struggle to faithfully recreate the spatiotemporal heterogeneity of human tumors. Additionally, natural products typically possess multi-component, multi-target characteristics. While this helps generate synergistic effects, it increases the difficulty of clarifying specific molecular mechanism, potentially leading to off-target effects or ambiguity in mechanistic elucidation.

### The gap in clinical translation

Despite abundant preclinical data, high-quality, large-scale Randomized Controlled Trials remain scarce. Most current evidence remains at the stage of cell and animal experiments, lacking human pharmacokinetic data and long-term toxicology evaluations. Drug-Drug Interactions when natural products are combined with chemotherapy or immunotherapy are not fully understood; blind combination may lead to reduced efficacy or increased toxicity. For instance, certain natural products with antioxidant properties might weaken the efficacy of chemotherapeutic drugs that rely on Reactive Oxygen Species (ROS) to kill tumors [327].

### Clinical trial evidence for np-based breast cancer therapy

Compared with extensive preclinical evidence, clinical studies of NPs in breast cancer remain limited and are largely confined to Phase I and Phase II trials, with virtually no Phase III evidence currently available [15, 328, 329]. Most published studies have focused on conventional endpoints such as safety, tolerability, pharmacokinetics, objective response, and quality of life, rather than direct evaluation of TME-related changes [328, 329]. Among the NPs investigated, curcumin has shown the most advanced translational progress: early-phase trials indicate that curcumin combined with docetaxel, paclitaxel, or standard chemotherapy is feasible and may improve response or quality-of-life outcomes in selected settings [330, 331]. EGCG has also entered clinical evaluation, mainly in the context of radiotherapy-associated toxicity, where it showed acceptable safety and some benefit in reducing radiation dermatitis [332–334]. Other NPs, including Panax ginseng, fucoidan, and artesunate, have thus far shown more value in supportive care, co-administration safety, or disease stabilization than in clear direct anti-tumor efficacy [335–337]. Overall, current data suggest that combination therapy is more realistic than NP monotherapy in breast cancer, but further progress is limited by poor bioavailability, inadequate standardization, small sample sizes, lack of subtype-specific stratification, and the absence of TME-oriented biomarkers in trial design [15, 328, 329, 331]. Therefore, more rigorous and biomarker-guided clinical studies are still needed to determine whether the TME-modulating effects observed preclinically can translate into meaningful clinical benefit.

### Future perspectives

To address the challenges outlined above, future research should move beyond empirical screening and adopt more precise, mechanism-oriented strategies for NP development in breast cancer. One important direction is the integration of multi-omics technologies, artificial intelligence (AI), and network pharmacology to identify subtype-specific and TME-oriented NP interventions. By combining transcriptomics, proteomics, metabolomics, single-cell sequencing, and spatial omics, researchers may better define immune, stromal, vascular, and metabolic TME phenotypes across breast cancer subtypes [11, 77, 78]. On this basis, AI-assisted network pharmacology can be used to predict potential NP targets, identify key signaling modules, and construct compound–target–pathway interaction networks [338, 339]. In parallel, molecular docking and molecular dynamics simulation can further refine the predicted interactions between NP components and candidate targets, thereby improving the prioritization of bioactive compounds and helping design rational NP combinations or NP-based combination regimens [338, 340]. Importantly, future studies should also strengthen target validation by integrating computational prediction with chemical proteomics, affinity-based target fishing, thermal proteome profiling, and other target deconvolution strategies, so that direct molecular targets and mechanism-relevant pathways can be confirmed experimentally rather than inferred solely from in silico analysis [341]. Such integrated approaches may also support patient stratification in future NP-based clinical trials by linking specific TME phenotypes and molecular signatures with predicted NP responsiveness, thereby facilitating a shift from empirical “casting a wide net” to more precise and biologically informed intervention [77, 338, 339].

The development of next-generation smart nanodelivery systems is essential to overcome the major pharmacokinetic limitations of many natural products. Future efforts should focus on constructing smart nanocarriers with TME responsiveness, including pH-, enzyme-, ROS-, and hypoxia-sensitive systems, in order to achieve site-specific release, prolonged retention, and deeper penetration at tumor sites [20, 127]. At the same time, biomimetic delivery platforms based on biological membranes, such as exosomes or cell membrane-coated nanoparticles, should be further explored to improve biocompatibility, immune evasion, and targeting efficiency [22].

To build high-fidelity preclinical models which better preserve the heterogeneity and TME characteristics of human breast tumors. Patient-derived xenograft (PDX) and organoid models, as well as more advanced co-culture and humanized systems, may provide greater translational relevance than conventional homogeneous cell-line models [34, 124]. These models are particularly valuable for evaluating the context-dependent and multi-target effects of NPs and for improving the predictive value of preclinical screening results [34, 124].

Finally, future studies should place greater emphasis on mechanistic exploration and biomarker-guided clinical trials for combination therapy. Natural products should be systematically evaluated in combination with immune checkpoint blockers (ICBs), anti-angiogenic agents, endocrine therapy, and novel chemotherapeutic drugs, with particular attention to their ability to reprogram immunosuppressive niches and promote the conversion of “cold” tumors into “hot” tumors [22, 78]. In addition, clinical trial design should become more rigorous and mechanism-oriented, incorporating molecular subtype, TME-associated biomarkers, pharmacokinetic-pharmacodynamic relationships, and patient stratification criteria to clarify the optimal timing, formulation, dosing, and combination regimens for NP-based interventions [77, 339]. This will be crucial for accelerating the translation of promising preclinical findings into clinically meaningful benefit.

In summary, natural products represent a valuable resource for remodeling the breast cancer tumor microenvironment. By overcoming challenges such as poor bioavailability, limited standardization, and tumor heterogeneity, and by integrating emerging technologies including multi-omics, AI, network pharmacology, molecular modeling, advanced delivery systems, and more predictive preclinical models, NP-based strategies may become an important component of future comprehensive breast cancer treatment, ultimately improving patient outcomes.

## Conclusion

In summary, the breast cancer TME is a highly dynamic and heterogeneous ecosystem that actively drives immune escape, stromal remodeling, angiogenesis, metabolic adaptation, therapeutic resistance, and metastatic progression. Within this context, natural products have emerged as promising modulators of the TME because of their structural diversity, multi-target pharmacology, and relatively favorable safety profiles. Current evidence indicates that natural products can remodel the breast cancer TME through multiple interconnected mechanisms, including induction of immunogenic cell death, enhancement of antigen presentation, repolarization of tumor-associated macrophages, attenuation of MDSC- and Treg-mediated immunosuppression, restoration of innate cytotoxic immunity, inhibition of CAF activation and extracellular matrix deposition, suppression of hypoxia- and VEGF-driven angiogenesis, and correction of dysregulated glucose, lipid, and amino acid metabolism. Together, these activities highlight the broad therapeutic potential of natural products not only as anti-tumor agents, but also as regulators of the tumor-supportive niche.

Despite these encouraging advances, the clinical translation of natural product-based strategies for breast cancer remains constrained by several challenges, including poor bioavailability, inadequate standardization, limited mechanistic resolution, and insufficient biomarker-guided clinical evidence. In addition, the marked heterogeneity of the breast cancer TME across molecular subtypes underscores the need for more precise and context-dependent evaluation of natural product activity. Future progress will require deeper integration of multi-omics approaches, spatial and single-cell technologies, AI-assisted target discovery, advanced delivery platforms, and more predictive preclinical models to clarify mechanisms, optimize formulations, and identify responsive patient populations. Overall, natural products represent a valuable and mechanistically rich resource for breast cancer therapy, and continued advances in translational research may establish them as an important component of future TME-oriented and precision-based treatment strategies.

## Data Availability

Not applicable.
